# Geometrically designed domain wall trap in tri-segmented nickel magnetic nanowires for spintronics devices

**DOI:** 10.1038/s41598-019-45553-w

**Published:** 2019-06-21

**Authors:** Farzad Nasirpouri, Seyed-Majid Peighambari-Sattari, Cristina Bran, Ester M. Palmero, Eider Berganza Eguiarte, Manuel Vazquez, Aristotelis Patsopoulos, Dimitris Kechrakos

**Affiliations:** 1 Faculty of Materials Engineering, Sahand University of Technology, Tabriz, 51335-1996 Iran; 2 Instituto de Ciencia de Materiales de Madrid, CSIC Madrid, Madrid, 28049 Spain; 3 Department of Physics, National and Kapodistrian University of Athens, Athens, 15772 Greece; 4 Department of Education, School of Pedagogical and Technological Education, Athens, 14121 Greece; 5 Present Address: Division of Permanent Magnets and Applications, IMDEA Nanoscience, Madrid, 28049 Spain

**Keywords:** Magnetic properties and materials, Electronic and spintronic devices

## Abstract

“*Domain wall traps*” have been engineered and well-exploited in nanostrips by creating a *geometrical* trapping site, e.g. a single notch along a stripe, compared to diameter-modulated (DM) cylindrical magnetic nanowires (NWs) where multi-segmented DM-NWs have been generally studied. Here, we report our systematic study on the magnetization behavior, domain wall structure and its nucleation/propagation in tri-segmented diameter-modulated Ni nanowires, *a simple system to investigate the magnetization reversal as function of segment geometry and lay-out order*. We find out that the magnetization behavior of single Ni DM-NWs exhibits the significance of positional ordering of thick and thin segments, distinguished by two distinct geometries including: *dumbbell-type* (type I) and *rolling pin-type* (type II). Based on experimental and theoretical simulations, it was evidenced that the wide-narrow junctions create trap sites for domain walls where the narrow segment restricts their motion. This type of geometrically engineered nanowires exhibit potential efficiency for future novel spintronic devices in particular when assembled in arrays of DM-NWs as a practical three-dimensional memory device.

## Introduction

In recent years, particular attention is paid to three-dimensional (3D) magnetic nanostructures as excellent candidates for magnetic storage device applications owing to the control of their magnetization reversal modes. The proposed racetrack memory device has been an impressive breakthrough for the application of magnetic nanowires as possible domain wall solid state storage devices^1^. For such advanced magnetic storage device it is crucial to consider magnetic nanowires with segments/parts/structures/notches having a sharp domain wall between two opposing thermally-stabilized domains^2^, where a simple robust switching controls the storage functionalities^3^. Therefore, the creation and propagation of a high quantity of domain walls along a single magnetic nanowire assembled in the form of three-dimensional array architectures is fundamental to produce this memory spintronic device.

Reports of *Cowburn* and co-workers^4,5^ on nanostripes or so-called planar magnetic nanowires, have pioneered the topic of domain wall nucleation and propagation for memory storage purposes. The design of the geometry in planar nanostripes has been proved to be very relevant for the injection and propagation of domain walls. Injection is usually achieved in a wide end of the nanotripe while their propagation and pinning is controlled through notches of reduced section. Indeed, a high aspect ratio segment is magnetically “soft”, with a low switching field and is utilized as a domain wall injection source. This is connected to a narrower segment, i.e. nanowire, whose switching field is greater called magnetically “hard” segment^6^. Thus, the junction of “soft” and “hard” segments localize the domain wall which will selectively propagate to the thin nanowire segment^7^. The realization of this idea has been generally exploited in different material systems such as CoNi/Pt^8^, Co/Pt^9–11^ and Permalloy nanostripes. The electron beam lithography and focused ion beam irradiation known as expensive nanofabrication methods have been used to create such magnetic nanostructures. Other fabrication techniques including focused electron-beam induced deposition (FEBD)^12,13^ and two-photon lithography (TPL)^14,15^ have also been employed to realise the domain wall mediated 3D nanowires for novel data storage devices. Electrodeposition has offered cheap and flexible method of fabrication of 3D magnetic structures^16^ with tunable shapes and sizes. Indeed, template electrodeposition of magnetic nanowires is a simpler and cheaper approach than the two other techniques mentioned, i.e. FEBD and TPL. More recently, *multi-segments* grown longitudinally with different geometries and aspect ratio has been realized by electrodeposition to produce diameter-modulated and/or multi-segmented cylindrical magnetic nanowires. In this way, Co^17^, Ni^18–21^, NiFe^22–24^, CoNi^25,26^, CoFe^27^, FeCoCu^28–31^ and Fe_2_O_3_^32^ diameter modulated nanowires have been investigated either inside the array or in individual form.

Ni NWs having segments with diameters and lengths ranging between 30–110 nm, and 1–40 µm, respectively, have been electrodeposited and their magnetic properties, including magnetization reversal and domain wall structures have been shown to be size-dependent^33–37^. Cylindrically *two-segmented* DM Ni_80_Fe_20_ NWs having alternating diameters of 250 and 500 nm but different segment lengths of 2.5 and 6.5 µm were studied by *Salem et al*.^23^. Their magnetization reversal was governed by the nucleation and propagation of a vortex domain wall. The junctions of segments serve the location of the generation of vortex domains which propagate towards the nanowire ends. This generally takes place to minimize the magnetostatic energy induced by the surfaces normal to the initial magnetization direction of the nanowires. *F*. *Tejo et al*.^33^ reported that the domain walls nucleate at the ends of the nanowires subsequently depinned and propagated along the wire. The angular dependence of coercive field is a factor by which the magnetization mode or mechanism is determined and has been well developed to study the magnetization modes of nanowires^30^. Provided that the magnetization reversal is taken place by the coherent rotation mechanism, the Stoner-Wohlfarth model^34^ is valid. This was confirmed by *Landeros et al*.^35^, *Aharoni*^36^, *Escrig et al*.^37^ and *Allende et al*.^38^ for nanowires and nanotubes. Furthermore, *Lavin et al*.^39^ developed a model for Ni nanowire arrays having a diameter of about 50 nm. They have mentioned that the nucleation and propagation of domain wall occurs via the transverse model. However, the experimental and micromagnetic studies of *Vilanova Vidal et al*.^40^ appeared to report single domain behavior in Ni nanowires switched by the vortex domain wall along the nanowires. Therefore, it may be concluded that the nucleation and propagation in magnetic nanowires is taken place by a *transverse* wall and a *vortex* wall for smaller and larger diameters, respectively. The critical value depends on the composition/anisotropies. It is noteworthy that several works have been reported on DM NWs very few compared to regular magnetic nanowires.

**Figure 1:**
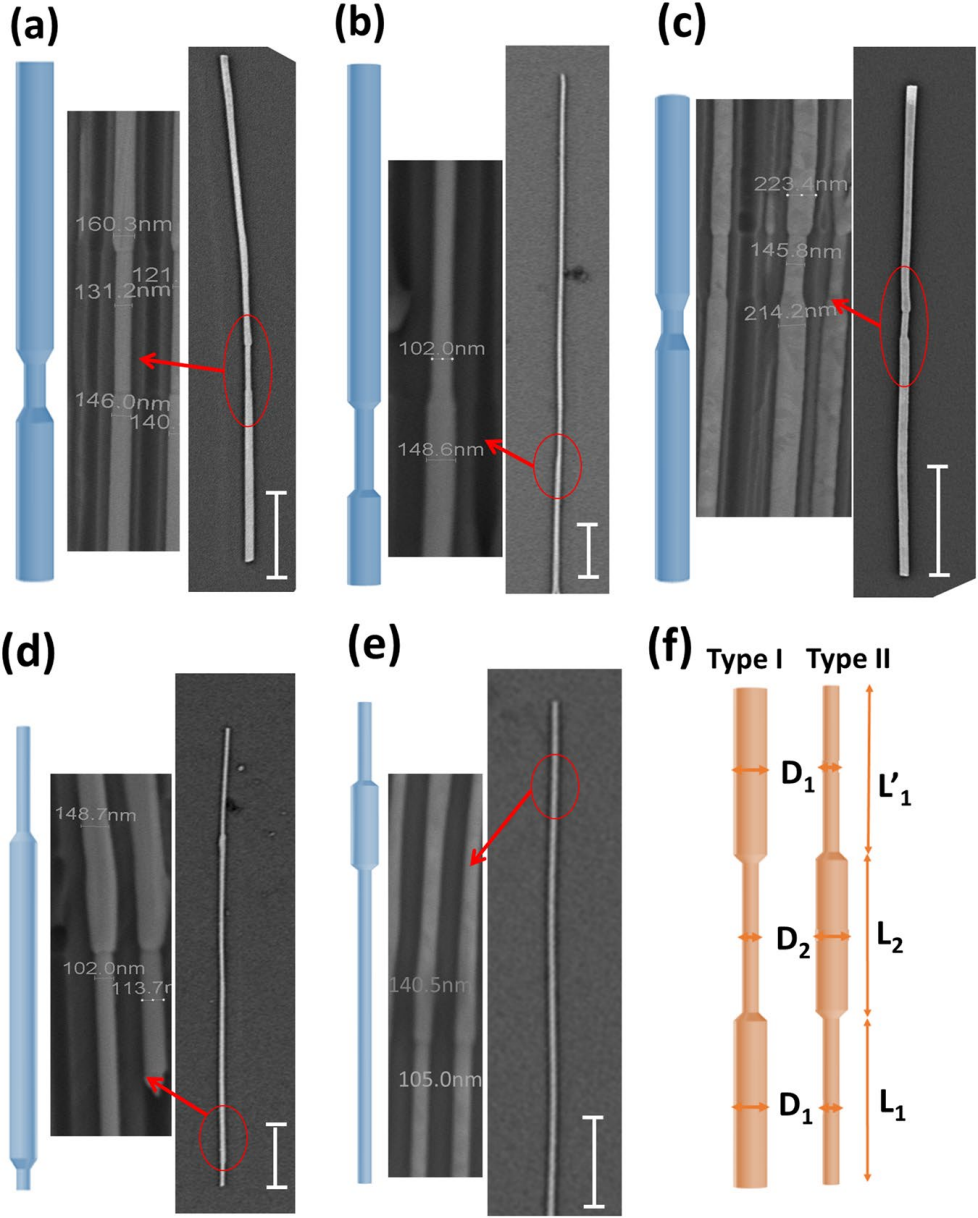
Schematic pictures (left panel) and corresponding SEM images (right panel) of individual Ni NWs nanowires on Si substrate of (**a**) I-1, (**b**) I-2, (**c**) I-3, (**d**) II-5 and (**e**) II-6. Panels in the middle show a magnified section of SEM images marked by a circle on corresponding right panels exhibiting the junction area with a diameter value measured. The scale bars shown in SEM micrographs in the right panels are 3 *μ*m. Panel (f) defines geometrical dimensions of Type I and Type II nanowires.

In this work, we study tri-segmented Ni cylindrical nanowires with two kinds of diameter segmentations, where our objective has been to exploit the mechanisms of nucleation of domain walls, their type and their propagation. For Ni NWs the shape anisotropy significantly is greater than the magnetocrystalline anisotropy, thus, we neglected the later in our assumptions. Thus, the former will solely control the magnetization reversal. We have simplified the nanowire structure into three segments with different aspect ratio. We discuss the effect of geometry in two cases: (i) Type I, *dumbbell-shape* nanowires, when the central segment has smaller diameter than the lateral segments, refer to Fig. 1a–c for the corresponding images and (ii) Type II, *rolling pin-*type nanowires, when the central segment has larger diameter, see Fig. 1d,e for the corresponding images. The effect of the geometry on magnetic properties of tri-segmented diameter-modulated nanowires, TS-DW-NWs, was investigated by means of experimental methods including magneto-optical Kerr effect (MOKE), magnetic force microscopy (MFM) and Monte-Carlo micromagnetic simulations. Such NW structures are anticipated to motivate the development of domain-wall mediated storage devices with strong control of magnetic domain wall creation and propagation.

## Results and Discussion

**Figure 2:**
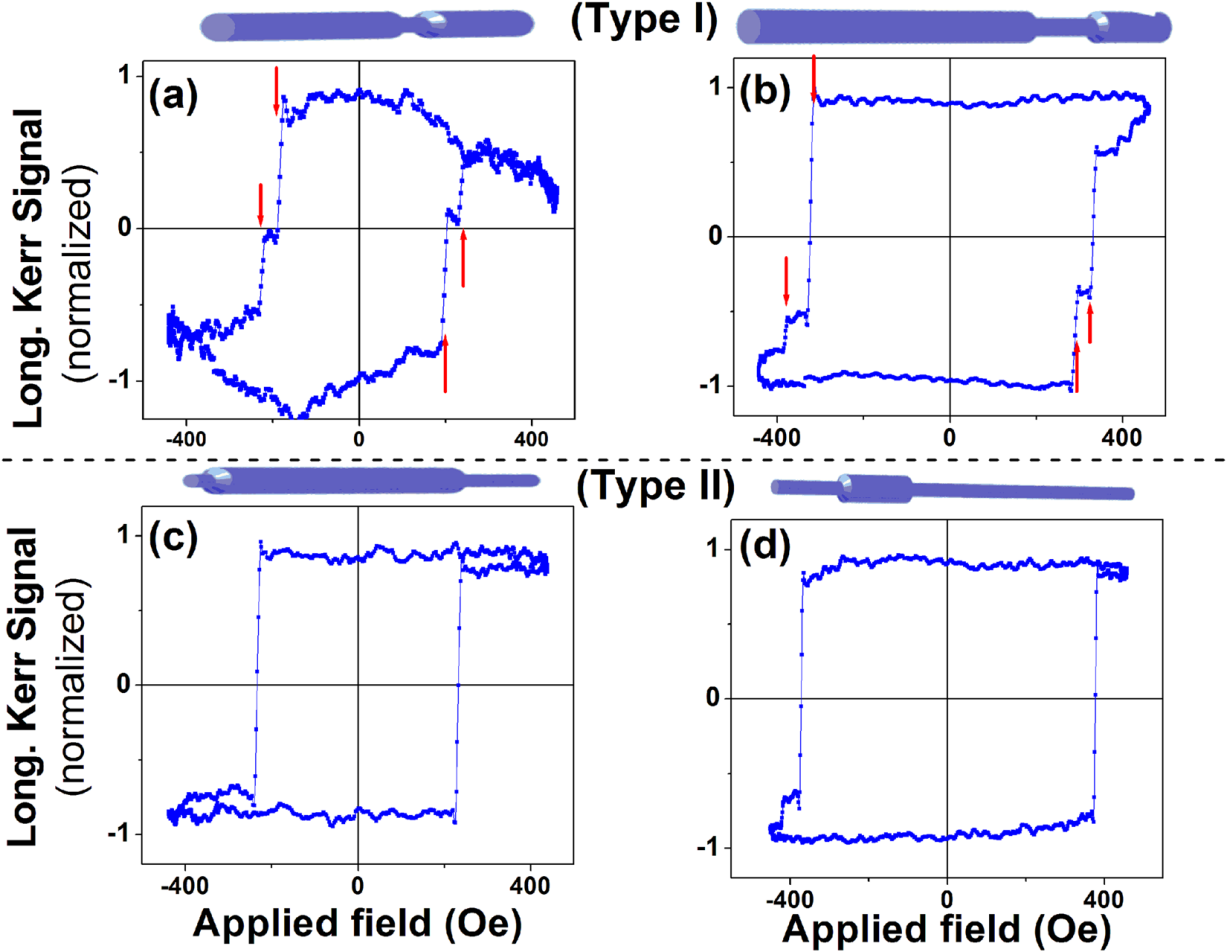
MOKE hysteresis loops of NWs taken at the center of Type I (top panel) and Type II (bottom panel). Schematic pictures of NWs as guides to eyes are inserted in top of the corresponding magnetization curve. The curves were reproducibly achieved by focusing the laser spot on the center of the nanowire.

Figure 1 shows schematic pictures and SEM images of fabricated diameter modulated nanowires. *Dumbbell-like* and *rolling-pin* tri-segmented nanowires having different thick (D_max_), thin (D_min_) and mean diameter (D_mean_) with high aspect ratio were successfully electrodeposited. D_max_ was in the range of 135–209 nm and D_min_ was in the range of 98–151 nm for different DM NWs studied. Full details of the dimensions of all NWs are shown Table 1. Figure 2 shows MOKE hysteresis loops of representative nanowires where the laser spot was focused on the middle of each nanowire. The MOKE hysteresis loops measured in a magnetic field which was applied parallel to the wire’s long axis. The MOKE loops were simultaneously averaged during the measurement by a factor of 800 to 1000 to optimize the signal to noise ratio. Totally, the longitudinal Kerr signal normalized to remanence is close to unity at 500 Oe (the maximum applied field). Such square or bi-stable loop with a single giant *Barkhausen* jump is typically observed for constant diameter nanowires^31,41,42^. The existence of only a jump (or a giant jump) indicates that a single domain wall propagates long distance in the wire even from one end of the wire to the other originating a single Barkhausen jump. In fact, it is relevant to the main objective of the present study since it allows us to conclude that the magnetization reversal is driven by the propagation of a single domain wall, not as due to random and uncorrelated movements of walls. However, there are different features for different types of NWs. A sharp transition is observed, similar to constant diameter nanowires, for Type II nanowires II-6 and II-5. However, a different behavior is observed for Type I NWs where two switching fields are identified. The switching fields in sample I-2 are better defined most probably due to the larger length of the segments.

**Table 1:** Anodization voltage and time employed for the fabrication of AAO templates and the resulting geometrical parameters of the nanowires.

Nanowires labels	L1 (μm)	L2 (μm)	L‘1 (μm)	D1 (nm)	D2 (nm)	D_mean_ (nm)
	t1(s)	t2(s)	t‘1(s)	V1(v)	V2(v)	V (μm3)
Type I-1	9.7 ± 0.5	1.6 ± 0.2	5.5 ± 0.1	159 ± 15	130 ± 8	144.5
	2650	30	500	100	130	0.32
Type I-2	6 ± 0.3	5.5 ± 0.2	23 ± 1.0	145 ± 13.0	98 ± 11.2	121.5 ± 10.5
	1360	20	1500	100	130	0.52
Type I-3	6.7 ± 0.1	0.67	6.1 ± 0.3	209 ± 18	151 ± 8	180
	1250	10	400	100	130	0.45
Type II-5	5.4 ± 0.5	15.1 ± 0.4	1.2 ± 0.1	106 ± 8	147 ± 13	126.5
	1690	1000	40	130	100	0.31
Type II-6	28 ± 3.5	6.98 ± 0.5	7.09 ± 0.1	104 ± 5	135 ± 15	119.5
	1700	600	30	130	100	0.39

Parameters are defined in Fig. 1f.

The presence of magnetization jumps in the hysteresis curves of diameter modulated nanowires was detected in periodically diameter-modulated FeCoCu nanowires^31^ and multi-segmented diameter modulated Ni nanowires^19^. However, we only here observe two distinct jumps or steps during magnetization whose reason has although not systematically been explained in detail before. The presence of such jumps has been ascribed to the presence of metastable magnetic states during the magnetization reversal correlated by the nature of the diameter modulation. The origin of such states was suggested to arise from the pinning of the magnetic domain boundary at the junction of diameter modulating segments leading to individual magnetization jumps when the wall depinned. In addition, earlier studies have highlighted the pinning sites created by micro-structural irregularities like crystal defects, roughness, and even magnetic impurities^13,25^. It is noteworthy to mention that loops in Fig. 2 are practically symmetrical for positive and negative fields. In the following, we have simplified the diameter modulated nanowire systems in only three segments in order to better understand the formation of such metastable magnetization states.

**Figure 3:**
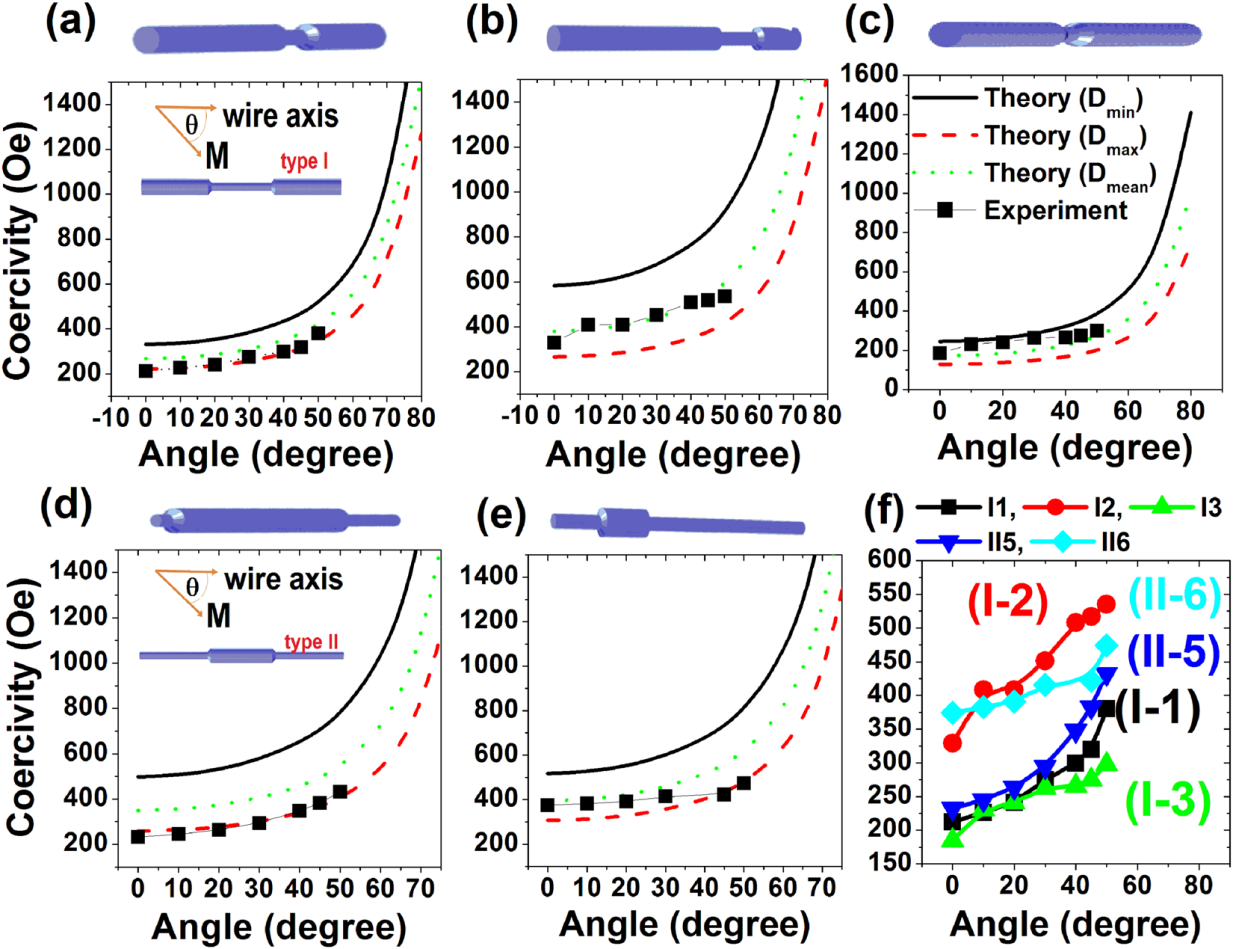
Angular dependence of coercivity (or the larger switching field) fitted by different values of segment diameters of Type I and II Ni NWs (**a**) I-1, (**b**) I-2, (**c**) I-3, (**d**) II-5, (**e**) II-6 and (**f**) integrated curves of different wires in one panel. Inset to panels (a) and (d) show schematically the angular configuration of two Types of NWs. Schematic pictures of each NW is shown in upper section of the corresponding panel. Legends of panels (a) to (e) are same only shown in panel (c).

The angular dependence of coercivity was used to establish mechanism nucleation/propagation of domain walls in our nanowires^30^. The angular dependence of coercivity, *H*_*c*_, is shown in Fig. 3 (additional details on the hysteresis loops can be found in the Supplementary Information). For all the nanowires, we observe a continuous increase of *H*_*c*_ with the angle. Note that for the highest angles, close to 90°, no data is given because of the limitations in the maximum applied field. As observed in Fig. 2, Type I nanowires exhibit a multi-step magnetization reversal revealed by sharp jumps on the magnetization curves, while Type II nanowires show a single giant *Barkhausen* jump. Any sharp jump on the magnetization reversal corresponds to a switching field, *H*_*sw*_. For Type I nanowires, where there are two switching fields corresponding to the jumps or “knees” on the curves along each branch, we have calculated *H*_*c*_ as the mean value of the switching fields. However, it is worth noting that the single switching field which appeared for Type II nanowires is directly proportional to the coercivity, *H*_*c*_. Thus, in Type II nanowires, *H*_*c*_ corresponds to the single switching field, *H*_*sw*_.

According to the angular dependence plots, the coercivity or switching field increases as a function of magnetization angle. Therefore, we find out that the magnetization reversal is controlled by the propagation of a vortex domain wall known also as the Bloch-point domain wall in magnetic nanotubes and nanowires^31,33,43^. For a vortex magnetization reversal mode, the angular dependence of the coercive field for an isolated nanowire with high aspect ratio is given by the expression^36^:

1 $${H}_{sw}=\frac{\frac{8{q}^{2}{A}_{exc}}{{M}_{s}{D}^{2}}(2\pi -\frac{4{q}^{2}{A}_{exc}}{\pi {M}_{s}^{2}{D}^{2}})}{\sqrt{\frac{16{q}^{4}{A}_{exc}^{2}}{{\pi }^{2}{M}_{s}^{4}{D}^{4}}si{n}^{2}\theta +(2\pi -\frac{4{q}^{2}{A}_{exc}}{\pi {M}_{s}^{2}{D}^{2}})co{s}^{2}\theta }}$$

where *H*_*sw*_ is the switching field, q^2^ = 1.08π for a cylindrical geometry^33^, A_exc_ is the exchange constant, M_s_ is the saturation magnetization, *θ* is the magnetization angle and D is the nanowire diameter. Provided that the magnetization of nickel at saturation, M_s_ = 485 emu.cm^−3^, we fit the experimental data of the switching fields obtained at different angles for different samples using Eq. 1. For all the modulated diameter nanowires, the fitting procedure was repeated for different D values including D_Max_, D_Min_, and D_Mean_ and the results are plotted in Fig. 3.

As evidenced from the plots, the experimental data are well fitted to the maximum diameter or wider segment for Type II NWs, particularly for small angles. This is a general behavior of diameter modulated magnetic nanowires with single giant *Barkhausen* jump where no abrupt small jump occurs and it follows the magnetization switching at the wider diameter segment, D_max_, as has been also shown also by *Palmero et al*.^31^. In contrast, that is not the case for Type I NWs and it is very likely that an imaginary mean value between wide and narrow diameters called mean diameter, D_mean_ rather closely fits to the experimental data, though some discrepancy exists. The discrepancy between the theoretical curves and experimental data of the coercivity for Type I NWs is observed very likely due to the small switching field in magnetization loops. The single value of coercivity considered here for Type I NWs (in Fig. 3**)** is the terminating switching field where the domain wall is essentially de-pined. The variation of the terminating switching field or the coercivity assumed here is far beyond the theoretical curve driven for nanowires with a straight diameter of D_max_ and is close to the curve plotted for D_mean_. This means that the magnetization takes places by a complex contribution from either wide or narrow segments. This feature of our data is more interesting which would exhibit metastable states in Type I Ni NWs. The increasing trend of the switching field or the coercivity is evident as a similar behavior for all types of nanowires studied, see Fig. 3f.

**Figure 4:**
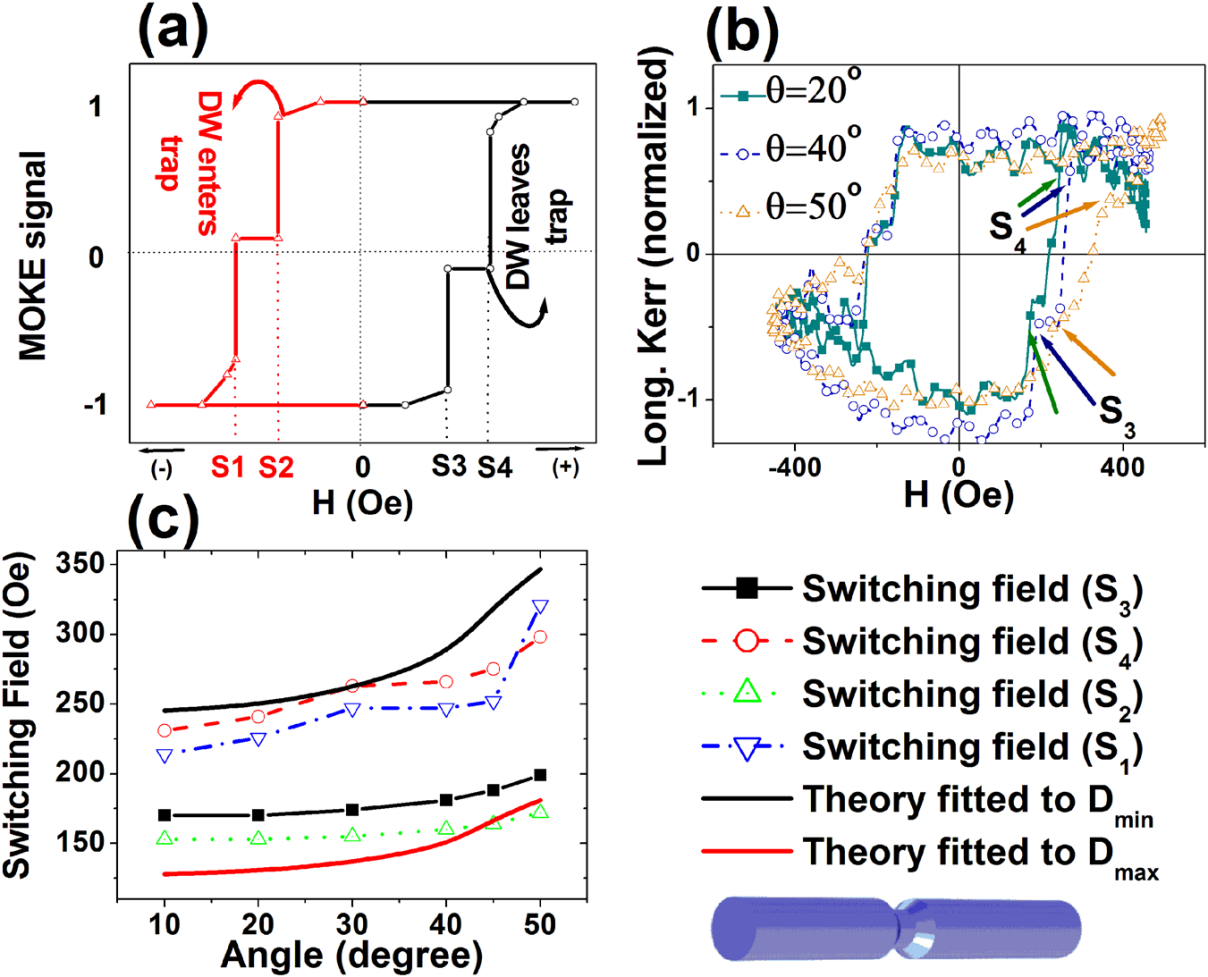
(**a**) Schematic representation of hysteresis loops of a typical Type I NWs. (**b**) Experimental MOKE magnetization curves measured at θ = 20°, 40° and 50° for NW Type I-3. (**c**) Comparison between experimental and calculated (Eq. 1) angular dependence of switching fields.

To understand the magnetization behavior of Type I nanowires, we have conducted a careful analysis on the angular dependence to interpret the existence of the abrupt jumps and clarify (i) how the magnetization process occurs, (ii) which segment, narrower or wider in diameter, controls the process and (iii) the switching sequence at reversal. Figure 4 shows a schematic MOKE hysteresis loop of Type I (sample I-3) nanowires having a symmetrical shape. The switching fields are marked by S1, S2, S3 and S4, where both S2 and S3 denote the small or initiating switching fields and both S1 and S4 are terminating final magnetization states (i.e. larger switching field) on each branch of the loop. These switching fields were collected from the experimental hysteresis loops of Type I-3 NWs measured at different angles in Fig. 4b, and compared to the theoretical values in Fig. 4c. We compare the two observed switching fields with the theoretical calculation according to Eq. (1) performed for the wider and narrower diameter segments.

With increasing the angle, the gap between the small and large switching fields becomes wider indicating the presence of a metastable state for Type I Ni NWs. There is a good agreement between the measured switching fields and the values calculated according to Eq. (1). The initial and final switching fields correlate respectively to the calculated values for the wider (D_max_) and narrower (D_min_) diameter. This indicates that the wider segment with a diameter D_max_ acts as the nucleation of domain wall as is always the case. Furthermore, an intermediate metastable state is created when a domain wall reaches the transition from the wider to the narrower segment until the applied field becomes high enough to overcome that barrier and it gets inside the narrower segment. Indeed, a similar phenomenon has been previously reported by C. Faulkner *et al*.^44^ in planar nanostrips where a notched region created by ion beam milling plays the role of a domain wall trap site. The domain wall nucleates at the ends of the nanowire where segments are wider and propagates in the small switching field until it reaches the trap and it leaves the trap at the large switching field. Thus, the study of domain wall movement may be controlled by many factors including geometrical trapping/releasing. This is of great interest for higher density magnetic random access memory systems where a method of reduction of cost and dissipation of storage energy is provided by using a “domain wall trap”^45^. In the case of Type II nanowires, where the central segment has the wider diameter, we observe a single Barkhausen jump. Therefore, we can assume that the domain wall nucleates at the ends of the nanowire and once it is de-pinned at the coercivity or switching field it propagates without any barrier at the diameter transition to the segment of wider diameter.

More detailed information on the influence of diameter modulating junctions at a submicron scale can be found in the Supplementary Information. There, the profile of the MOKE signal was recorded in each individual nanowire at different positions along the nanowires’ length. We have definitely observed significant differences between two types of nanowires based on the local loops recorded at different locations. Here, we only show data for two representative samples of the two different types of NWs. On one hand, nanowires Type II-6 exhibit the general behavior of magnetization discussed in above which remains unchanged along the nanowire. Little differences among the hysteresis loops are in the error range of the measurement techniques (for instance variation of the coercivity about 10 Oe). On the other hand, as mentioned before, “knees” are present on the magnetization curve of type I NWs. The hysteresis loops were measured at different positions across the length of the nanowire. At the left side end, away from the segments junction, there is only one jump in one branch of the hysteresis loop. But two sharp jumps appear by approaching to the junction where they are both seen in either branch of the hysteresis loops. These experiments reveal the importance of the junction geometry distribution on the domain wall nucleation and propagation.

**Figure 5:**
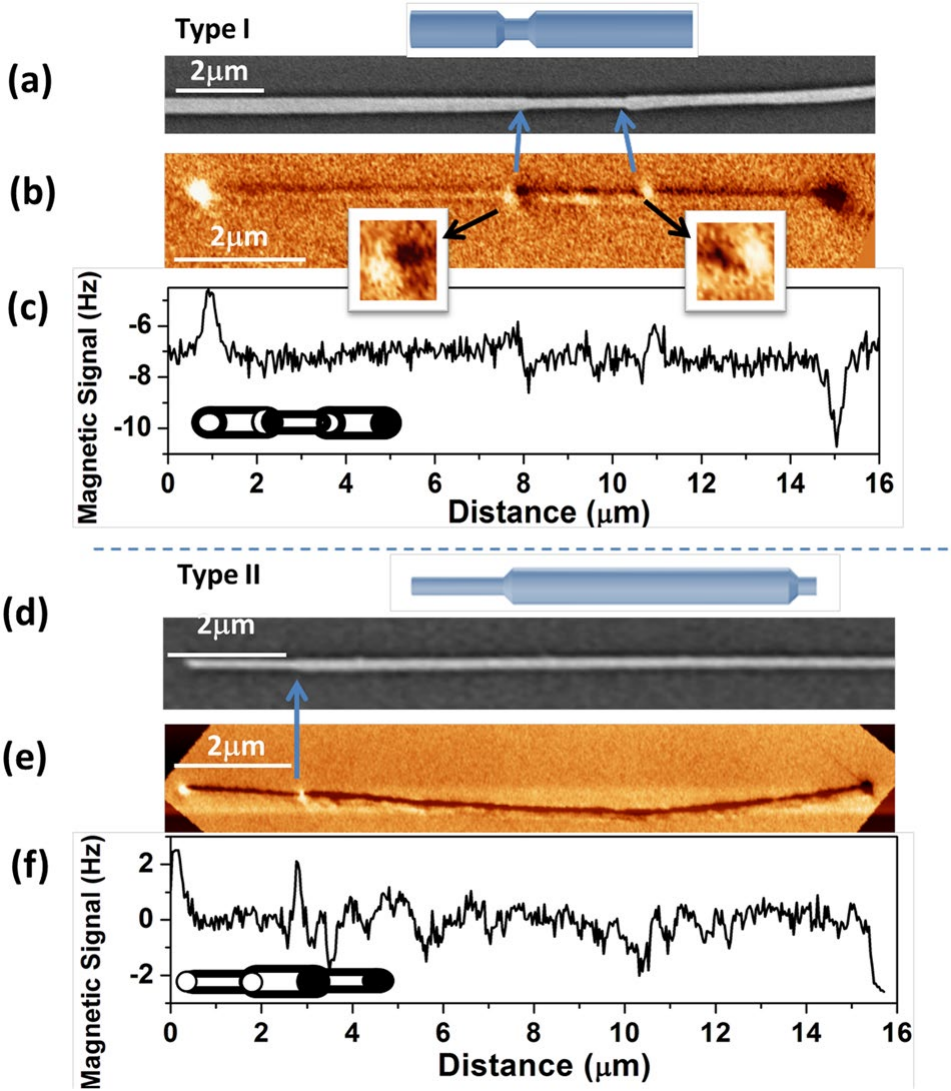
MFM images of Type I (I-1) (top panel) and Type II (II-5) nanowires (bottom panel). (**a**,**d**) SEM images, (**b**,**e**) MFM images and (**c**,**f**) magnetic signal profiles along the nanowires. Insets to (**c**,**f**) are the schematic representation of domain structures.

MFM was employed to image the magnetic configuration particularly at the junction of segments. Figure 5 shows SEM and magnetic images of Type I and Type II nanowires at remanence state. Dark and bright contrasts due to the presence of accumulated magnetic charges are observed at the ends of both types of NWs. In addition, MFM image in Fig. 5(b) displays additional less intense contrasts at the modulation junctions in Type I NW. The contrasts magnified in Fig. 5b are reversed through the central segment as is schematically illustrated in the inset to Fig. 5c. While some effect of the stray field emanating from the standard magnetic tip could not be completely discarded^46^, according to our micromagnetic simulations that will be discussed later we conclude that the magnetization starts with the nucleation of a domain wall from thick segments and reaches the junction from either side. This might be a reason for the creation of such contrast spot on the MFM images. However, this is not the case for Type II NWs which only exhibits the most usual behavior of constant diameter NWs where the magnetic poles with opposite contrast are observed only at both ends of the nanowire. That typical dipolar contrast is commonly ascribed to the axial magnetization, and the geometry effect can be clearly observed in accordance with previous research work on FeCoCu bamboo-like nanowires by *E*. *Berganza et al*.^30^.

Over all based on our experimental data presented so far, our finding reveals that Type I nanowires have a distinct effect on the magnetic configuration and domain wall structure. All NWs present MFM images that are compatible with the single domain configuration, but only type I NWs exhibits additional local spin divergence induced at the site of narrow-wide junction. This may explain the effect of the junctions on the pinning and depinning of the domain wall as discussed in above. On the other hand, the presence of a vortex (or a system of vortices)^47^ could be expected in this type of nanowires as predicted by our angular dependence magnetization measurements. However, MFM is hardly sensitive to the formation of pseudo-vortex in nanowires of similar diameter as reported before, to this pseudo-vortex due to the lack of stray field^30^.

**Figure 6:**
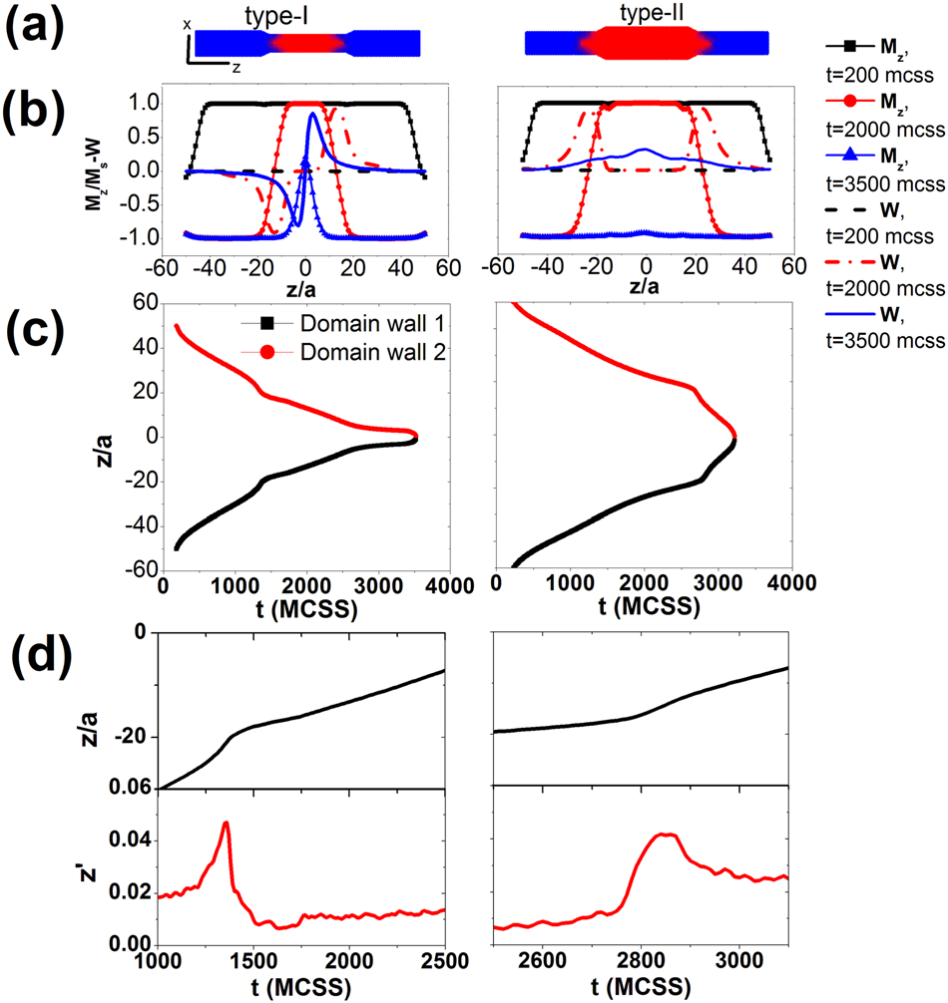
(**a**) Spin configuration of Type I and II nanowires when the domain wall approaches the junction. A color code is used to indicate the spin direction, namely red for spin up and blue for spin down. (**b**) Magnetization profile (marked lines) and vorticity profile (dashed line) at various instants during the reversal for Type-I (left) and type-II (right) nanowires. The junctions region for both types extends from z = −17a to z = −21a and from z = 17a to z = 21a. **(c)** Position of the domain wall center at different times for type-I (left) and type-II (right) nanowires. Red (black) line indicates the DW propagating from top-end (bottom-end) towards the center of the nanowire. Notice the change in average velocity when a domain wall enters the junction. (**d**) Instant position of DW around the time it reaches the junction (upper panel) and corresponding instant velocities (lower panel). A sudden drop of the instant velocity to almost zero value for a short time (1500–1750 MCSS) indicates a temporary pinning of the DW in the junction region of type-I NWs. The effect of DW pinning at the junction region is not observed in type-II nanowires. MCSS stands for standard abbreviation for Monte Carlo Steps per Spin.

In order to obtain deeper insight into the microscopic reversal mechanism, we have performed Monte Carlo simulations on the magnetization reversal (*see Methods*). To analyze the domain wall (DW) dynamics in a Type I and II nanowires, we started the simulation with the system at positive saturation magnetization (M = + M_s_) and a reverse field (H~ -H_c_) is applied along the wire axis (z-axis). The applied (reverse) field is kept constant close to the coercivity value, which is different for the two types of NWs. Experimentally the applied field sweep is slow enough with respect to the DW motion. This would allow us to consider it constant in our simulations. In Fig. 6(a,b), we show instants of the magnetization profile and the vorticity profile when the domain wall approaches the junctions and as the variation plots, respectively. The vorticity is defined as $w(z)=\frac{1}{{N}_{z}}\sum _{i\in z}{\hat{r}}_{xy,i}\times {\hat{S}}_{xy,i}$ and parameterizes the presence of a vortex on a certain layer along the NW axis, namely, w = −1(+1) at z_0_ when a vortex with (counter) clockwise vorticity exists on layer z_0_. These data indicate that two vortex domain walls (VDW) form at opposite ends of the nanowire and propagate towards the center of the wire, where they eventually merge leading to complete magnetization reversal. Notice that our model of the tri-segmented nanowires supports vortex domain walls (VDW) in both the narrow and the wide regions of the nanowire, which is in accordance with our experimental observations by the angular dependence data where the diameters of all samples are above the critical value for vortex formation.

In Fig. 6(c) we plot the time-evolution of the domain wall position. What is interesting is that the velocity of the VDW drops when it enters the narrow region, as seen from the change of slope. In Fig. 6(d) we focus on the dynamics of VDW as it approaches the junction between different segments. In both types of NWs an overshoot of the instant velocity is observed, which is attributed to local softening of the material caused by the reduced coordination of moments in the junction. Indeed, when a cubic discretization is used to describe a smooth variation of diameter, inevitably the diameter variation is described numerically in discrete steps. Then some cells right in the transition region will have fewer than 6 nearest neighbors. These are “softer” than the others, i.e. the local exchange field is weaker, which therefore supports higher instant DW velocity. As soon as the DW exits the junction region we observe for the type-I NW a nearly vanishing velocity for a short period of time (pinning effect) and then the DW velocity is stabilized to a lower value, as expected since the DW velocity in the narrower area is smaller compared to the velocity in the wider area. In type-II nanowires, we observe a stabilization of domain wall velocity to higher values as the domain wall enters the wide area, but no accompanying delay of the DW in the junction region is seen. This behavior indicates that domain wall pinning occurs only in the wide-to-narrow junction. It is worth noting that during the simulation for DW propagation, the applied field is kept constant (H~Hc). Type-I and II have different Hc values (see Supplement). In this sense, different reverse fields are applied in different MW types. These results we show in Fig. 6c,d. Notice that, the simulated M-H loops of either type of DMNW do not show a discontinuity (“knee”) (see the Supplementary Information) (Fig. 3S(c)), a result which is also in accordance to previous micromagnetic calculations of DMNW with similar diameter ratio^48^. The delay of domain wall propagation at the wide-to-narrow junction is captured by the MOKE measurements due to the small measuring time scale.

Based on our experimental and theoretical results, we exploited the magnetization reversal mechanism of Ni NWs that takes place by the propagation of a vortex domain wall with a net magnetization component appearing in the xy plane perpendicular to the wire axis. We mention here that the geometrical restrictions when we encapsulate a narrower segment between the two wider segments would damp the magnetization reversal process at the junction site as a pinning site or domain wall trap site with different energy potential imposed to the reversal system as an energy barrier^49^. This effect is associated with the formation of sharp jumps or “knee” on the magnetization curves developing bi-stable or meta-stable states in the magnetization reversal. This type of geometrically engineered nanowires could be realized as novel domain wall mediated spintronic data storage devices. In particular, one of the challenging issues for the implementation of such nanowires into application is to create high density of domain walls. One solution is to fabricate three-dimensional architectures using alumina nanoporous templates as has been realized in^50^ which can be filled with our TS-DM NWs. Our nanowire arrays can be proposed to represent an ensemble of the tri-segmented nanowires inside the templates towards high density 3D domain wall mediated memory devices^51^, though they need to be studied from magnetic properties in array form whose magnetization behavior relies on the geometrically induced magnetic interaction and stray fields emanating from the modulating segments inside the array^19^.

## Conclusion

We have successfully fabricated and studied the magnetization and domain wall structure of individual tri-segment diameter modulated nickel nanowires. Based on MOKE measurements, MFM and angular dependence of magnetization, we conclude that vortex-domain wall nucleates and propagates along the nanowires. It was successfully demonstrated that by engineering the geometry and dimensions of modulating segments, the entire magnetization reversal as well as the initial reversal event can be localized on the junction of thicker-thinner segments. For a peculiar geometry when a segment with narrower diameter is encapsulated between two segments with wider diameter, Type I nanowires, bi-stable or meta-stable states appear which may act as domain wall trap site as a potential candidate of domain wall mediated spintronic memory devices. This is bi-stable domain wall mediated switching at the junction site. This structure is of great technological importance for data storage applications. Monte Carlo simulations support the presence of vortex domain wall structures and show that the meta-stable states are a consequence of fast magnetization dynamics which could be an origin of pinning/depinning sites of domain walls. The dynamics of DW motion achieved by Monte-Carlo simulations directly provides further details of our idea of DW trap in Type I nanowires. This was exhibited by a delay in the dynamics of domain wall motion at wide to narrow junctions, which in fact was shown in the hysteresis loops by a switching field. The arrays of such tri-segmented diameter modulated nanowires embedded in highly ordered nanoporous alumina templates seem to assume a suitable three-dimensional architecture for memory device applications.

## Methods

### Sample preparation

High purity aluminum (Al) foils (99.999 wt. %) were used as the substrate to fabricate highly ordered diameter-modulated nanopores inside the anodic alumina oxide, AAO, templates using pulsed hard anodization. Al foils were firstly immersed in KOH (200 g/l) and HNO_3_ (50%Vol), respectively. Then, they were electropolished in a rigorously stirred electrolyte composed of 60% perchloric acid and ethanol with 1:4 volume ration under a constant voltage of 20 V at a temperature of 0 (±2)°C for approximately 15 min, to achieve a highly smooth and mirror-like bright surface. Potentiostatic pulse anodization was subsequently carried out in an electrolyte containing 0.3 M oxalic acid and 5 ml ethanol at 0 (±2)°C.

The Al substrate at the bottom of the AAO templates was firstly removed by immersing it into a solution containing 6.8 g CuCl_2_, and 100 ml HCL in 200 mL H_2_O for about 20 min at ambient temperature. Pore widening was then carried out in phosphoric acid 5%wt for 80 min at 32 °C. Prior to the electrodeposition of nanowires, a gold layer with an average thickness of 200–250 nm was sputter-coated on one side of the AAO template making it conductive and to serve as electrode for direct current (DC) electrodeposition. Then, a gold layer was deposited at the bottom of pores by DC electrodeposition at 2.4 V. The potentiostatic electrodeposition of nickel was carried out at room temperature into the AAO templates as the working electrode using a conventional three-electrode electrochemical cell. A Platinum net was used as the counter-electrode. A saturated silver/silver chloride (Ag/AgCl) was employed as the reference electrode. The electrodeposition electrolyte consisted of 200 g/l NiSO_4_, 40 g/l NiCl and 0.5 M boric acid at an average pH of about 4. Current transients were recorded during the electrodeposition using a computer controlled data acquisition system and the deposition time was varied depending on the filling time of the pores. The Supplementary Information contains additional details about the typical anodization voltage waveform and the corresponding current transient employed to produce different geometries and types of diameter modulated anodic aluminum oxide templates. It also reports the anodization parameters used to grow different types of diameter modulated AAO templates. Tri-segmented DM-Ni nanowires with different segment aspect ratio were electrodeposited into pulse-anodized AAO templates.

Nanowires were released from the template to study their individual behavior following several steps: (1) dissolution of AAO templates in a solution of chromic (0.2 M) and phosphoric acid (0.4 M), (2) dilution and replacing that with deionized water in a centrifuge for several times, (3) substituting deionized water with ethanol in a centrifuge and (4) releasing a droplet of final solution containing nickel nanowires on a silicon grid.

Scanning electron microscopy (SEM-Nova Nano 230) was used to study the morphology, geometry and position of nanowires.

### Magnetic measurements

Magnetic measurements of the arrays of nickel nanowires embedded in the AAO templates were carried out by a vibrating sample magnetometer (model KLA-Tencor EV7) at different angles between the applied field orientation and the plane of the template, from 0 (in-plane) to 90 (out-of-plane) degrees.

A magneto-optical Kerr effect (MOKE) instrument (NanoMOKE-TM 2) was used for magnetic measurements under maximum applied field of 500–600 Oe. A laser spot with a nominal diameter of 3 μm and a wavelength of 658 nm and power of 7.5 mW was employed in the instrument. The MOKE magnetization measurements were carried out by applying an external magnetic field along the wire axis. The curves obtained by averaging the hysteresis loops during the process 800 to 1000 times to achieve the best signal to noise ratio. Also, the laser spot with the size of one diameter modulation was focused on the nanowire at different positions. Angular MOKE measurements were done by applying magnetic fields at different angles with the axis of the nanowire from parallel (0°) to perpendicular (90°) as long as clear signal are received in steps of 10°. Longitudinal local magnetization hysteresis curves were recorded from one end of the nanowire to the other end with a step of 3–5 μm. The MOKE signal was normalized to the received one under the maximum applied field at each position.

The domain structure of individual nanowires dispersed on silicon substrate was imaged by atomic/magnetic force microscopy system (AFM/MFM) from Nanotec Electronica. A commercial CoCr standard magnetic tip was used to examine the magnetic domain structure of the nanowires individually dispersed on silicon substrate.

### Numerical simulations

To model the magnetic properties, cylindrical nanowires along the z-axis were discretized by a cubic lattice with cell size a^52^. The diameters of the segments are D_1_ = 16a and D_2_ = 24a, in accordance with the experimental diameter ratios (D_2_/D_1_ ≈ 1.40–1.50). The regions connecting the narrow and wide part of the DMNW have length L = 4a and are approximately described by a linear variation of diameter. The sequence of lengths of the three segments is 30a-40a-30a for both type-I and type-II NWs (Fig. 3S). The region of varying diameter (junction) extends along the wire axis from z = −17a to z = −21a and z = 17a to z = 21a, for both NW types. The total energy of the model system reads $E=\sum _{i}{E}_{i}$, where the on-site energy ${E}_{i}=-\,\frac{1}{2}J\sum _{ < i,j > }{\hat{S}}_{i}\cdot {\hat{S}}_{j}-\frac{1}{2}g\sum _{i,j}{\hat{S}}_{i}\cdot {\overleftrightarrow{D}}_{ij}\cdot {\hat{S}}_{j}-H\sum _{i}{S}_{iz},$ with *i*, *j* the site indices, *J* the exchange energy, *H* the Zeeman energy due to external field, *g* the dipolar interaction strength and ${\overleftrightarrow{D}}_{ij}$ the dipolar coupling tensor. In order to achieve the computational efficiency, the dipolar energy term is considered in an embedded cluster approximation having a cluster radius *r*_0_ = 5*a*^53^. We use micromagnetic parameters for Ni^54^, namely the exchange stiffness A = 1.3∙10^−11^ J/m and saturation magnetization M_s_ = 7.6∙10^5^ A/m and a discretization cell a = 5 nm. The isothermal hysteresis loops are simulated using the Metropolis Monte Carlo algorithm with a fixed spin-cone aperture (*θs* ≈ 3°) and 5 × 10^3^ Monte Carlo steps per spin (MCSS) for thermalization followed by 10^4^ MCSS for calculations of thermodynamic quantities. All simulations are performed at low temperature (*T/J* = 10^−3^).

## Supplementary information

Supplementary document
