# Supplementary material for: Geometrically designed domain wall trap in tri-segmented nickel magnetic nanowires for spintronics devices
Source: Sci Rep. 2019 Jun 21;9:9010. doi: 10.1038/s41598-019-45553-w (PMC6588627; doi:10.1038/s41598-019-45553-w)
Supplement: Supplementary file 1 — Supplementary document [file 41598_2019_45553_MOESM1_ESM.docx]

**Supplementary Information**

**Geometrically designed domain wall trap in tri-segmented nickel magnetic nanowires for spintronics devices**

Farzad Nasirpouri^1,*^, Seyed-Majid Peighambari-Sattari^1,2^, Cristina Bran^2^, Ester M Palmero^2,§^, Eider Berganza Eguiarte^2^, Manuel Vazquez^2^, Aristotelis Patsopoulos^3^ and Dimitris Kechrakos^4^

1. *Faculty of Materials Engineering, Sahand University of Technology, Tabriz, Iran.*
2. *Instituto de Ciencia de Materiales de Madrid, CSIC Madrid, 28049, Spain.*
3. *Department of Physics, National and Kapodistrian University of Athens, Athens, 15772, Greece.*
4. *Department of Education, School of Pedagogical and Technological Education, Athens, 14121, Greece.*

*-Corresponding author: nasirpouri@sut.ac.ir

**Experimental Methods and Results**

Figures 1S-a and b shows the anodization curves. In general, pulse-voltage anodic oxidation waveforms consist of an initial rapid sweep of voltage up to 80 V at a rate of 1.6 V/s followed by a constant voltage of 80 V for 400 s. The duration of each step was adjusted according to the structure of the AAO templates inquired. The current transients recorded during the pulsed anodization exhibited a relevant behavior as is explained here: In the first voltage pulse, the current initially rises up to a peak followed by a drop. After the second voltage pulse applied the current rises again up to a constant value showing a plateau on the graph. Increasing of time and voltage sweeping rate increases the length of first and second segments of nanopores in template. It should be considered that current peak occurring at each pulse is decreased continuously with respect to the previous pulses. On the other hand, the thickness of the barrier layer is increased whose resistance probably enhances after each pulse. Figure 1S-c shows a typical current transient curve recorded during the electrodeposition of nickel nanowires at a potential of 1.1 V Ag/AgCl into the diameter modulated AAO templates. The required time for filling of pores depends on the modulating diameters and their length as marked by kinks on the curve. Two current drops are visible on the current transients recorded for all of the nanowires that we fabricated. The two jumps may be described by the change of growth surface area exposed for electrodeposition at the smaller modulated segments. The first drop takes places for a shorter time whereas the second one is consistent with the diameter and lengths of modulated segments. Current is increased due to start of over-growth on top surface of template at the end of electrodeposition.


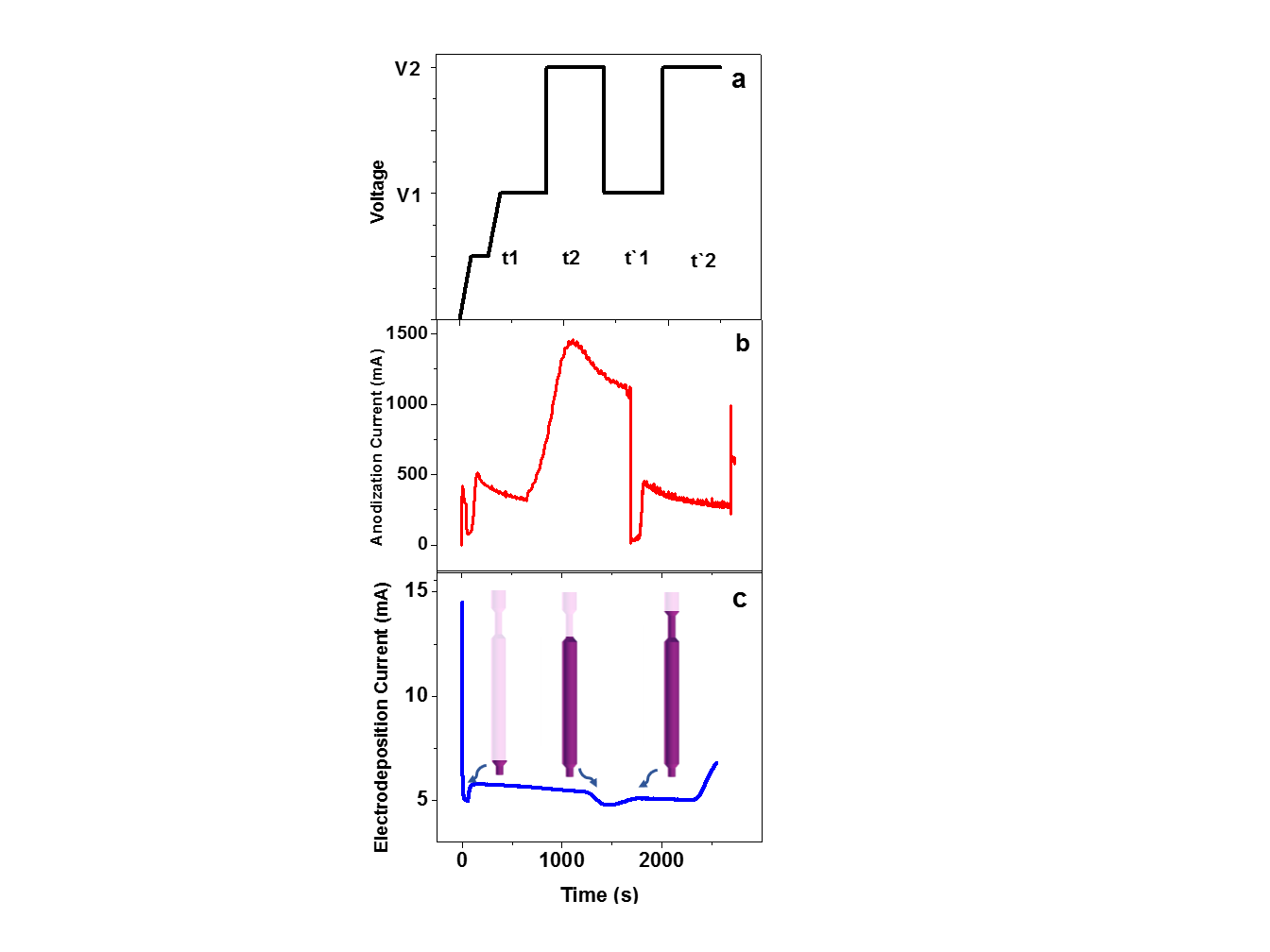


**Figure 1S.** (a) A typical voltage waveform and (b) corresponding current transient recorded for anodic oxidation diameter modulated nanoporous AAO template. (c) a typical current transient recorded during potentiostatic electrodeposition of nickel from an electrolyte containing 200 g/L NiSO4 .H2O 40 g/L NiCl and 0.5 M boric acid at a potential of 1.1 V Ag/AgCl.

The lengths and diameters of each Ni segment are shown in **Table 1S**. Length of each segment of the Ni nanowire is different as mentioned in Table 1S. It is to note that the density of pores and inter-pore distance remain unaffected by changing the anodization parameters including pulse time and voltage. However, the total thickness of templates and the length of first and second segments are increased and the diameter of them is decreased. The electrodeposition was controlled to eliminate any overgrowth happening on templates.

Pore diameters were solely adjusted by tuning the voltage. The temperature of pore widening solution has influenced the pore diameter as well. For DM-NWs, we have defined diameter values including: maximum diameter (D_Max_), minimum diameter (D_Min_), and mean diameter (D_Mean);_ Maximum diameter is the diameter of thicker (wider) segments and minimum diameter is the diameter of thinner (narrower) segments. D_Mean_ is the arithmetic mean, the sum of the diameters divided by number of segments. Based on their geometrical parameters, we have categorized them into two groups named type I and II. Type (I) is called *dumbbell-like* nanowires which have a segment with smaller diameter encapsulated between the two segments with a large diameter. In contrary, type (II) called *rolling-pin* like nanowires have a segment with a larger diameter encapsulated between two segments with a smaller diameter. The dumbbell-like nanowires (Type I), as function of the length and diameter of the segments, are divided in I-1, I-2 and I-3, while the rolling-pin nanowires (Type II) are divided in II-1 and II-2 (see **Table 1S**).

**Table 1S**. The angular dependence measured by the hysteresis loops of different Ni DM NWs.

| Nanowires | Magnetization Angle (Degree) | | | | | |
| --- | --- | --- | --- | --- | --- | --- |
|  | 10 | 20 | 30 | 40 | 45 | 50 |
| **I-1** | 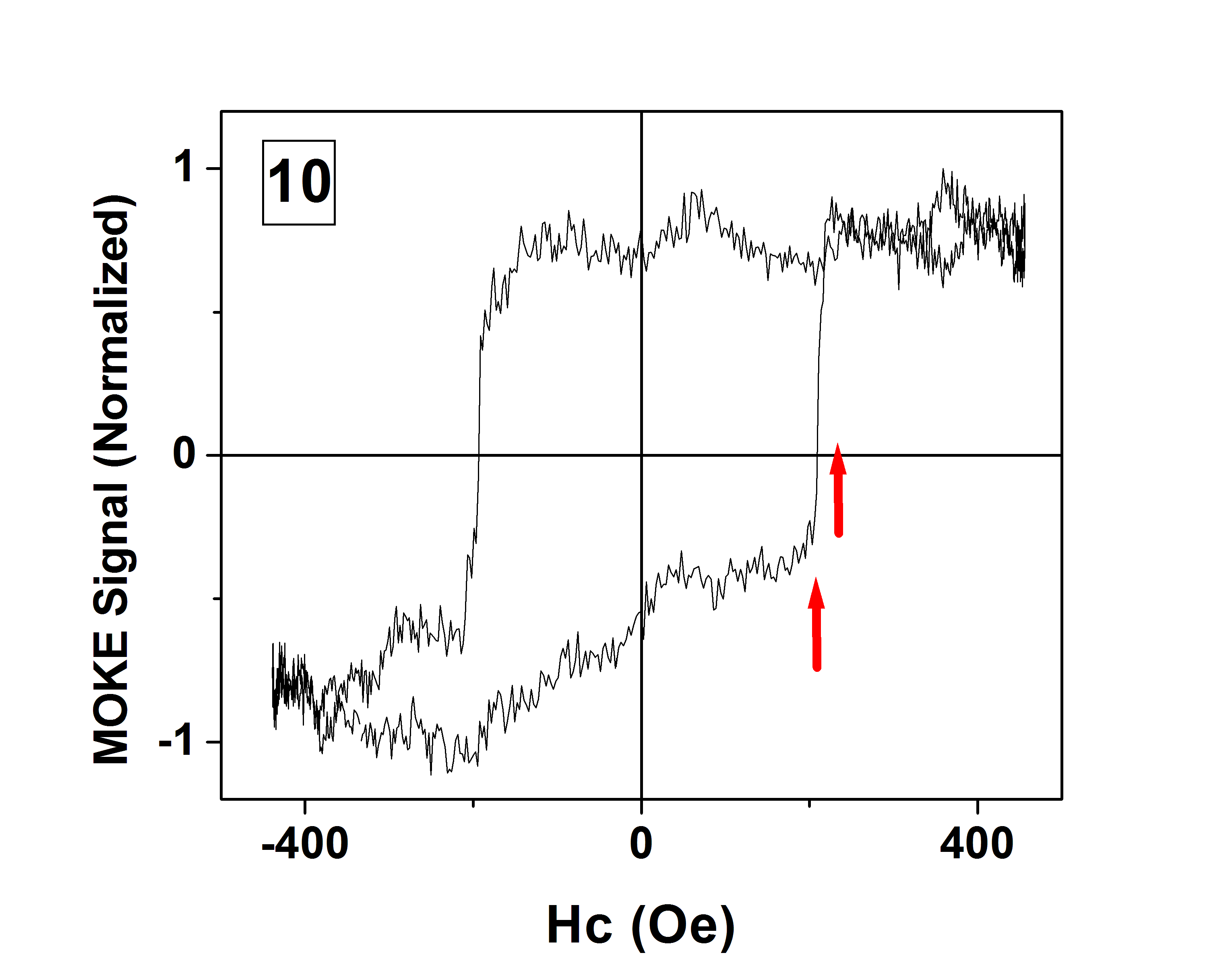 | 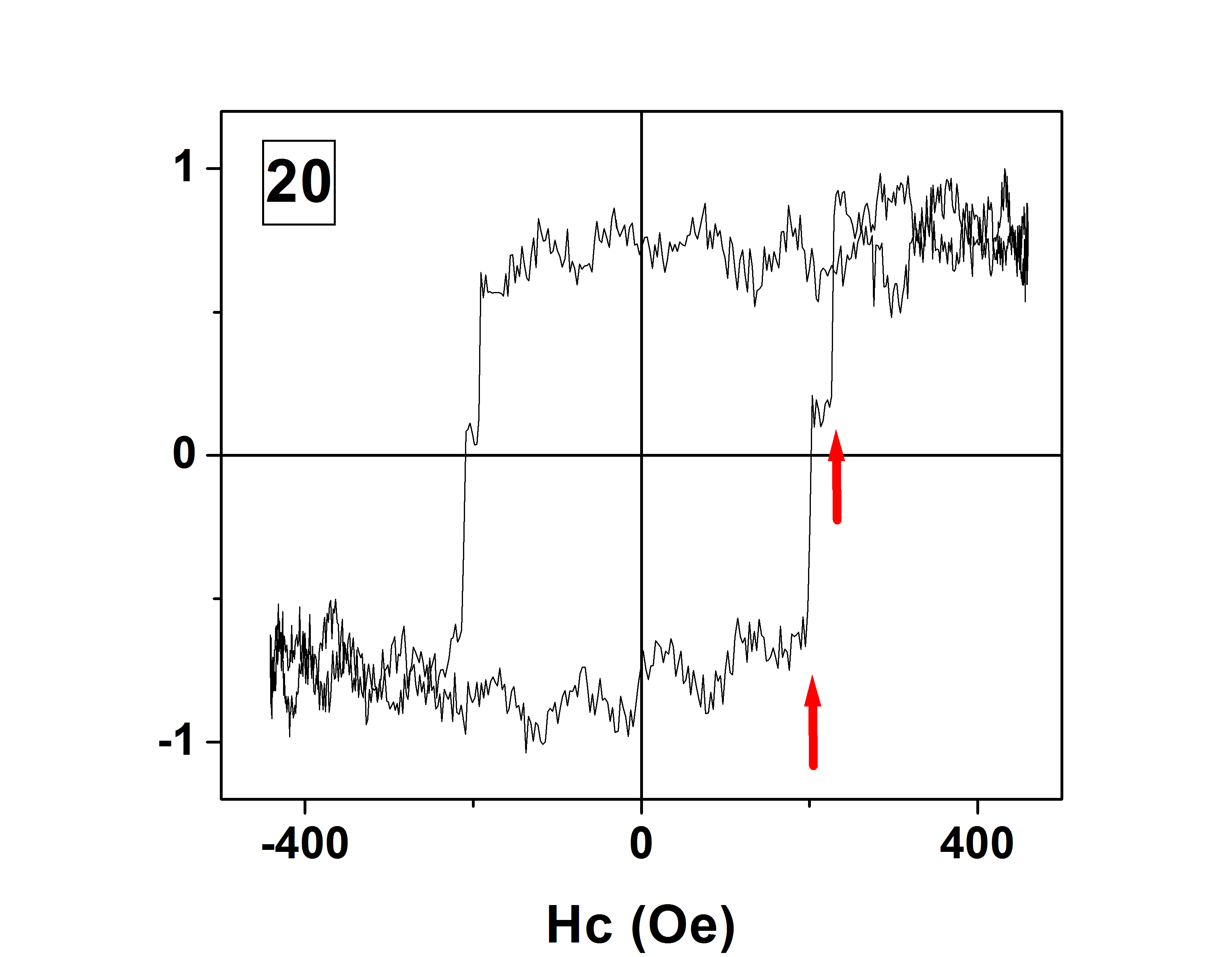 | 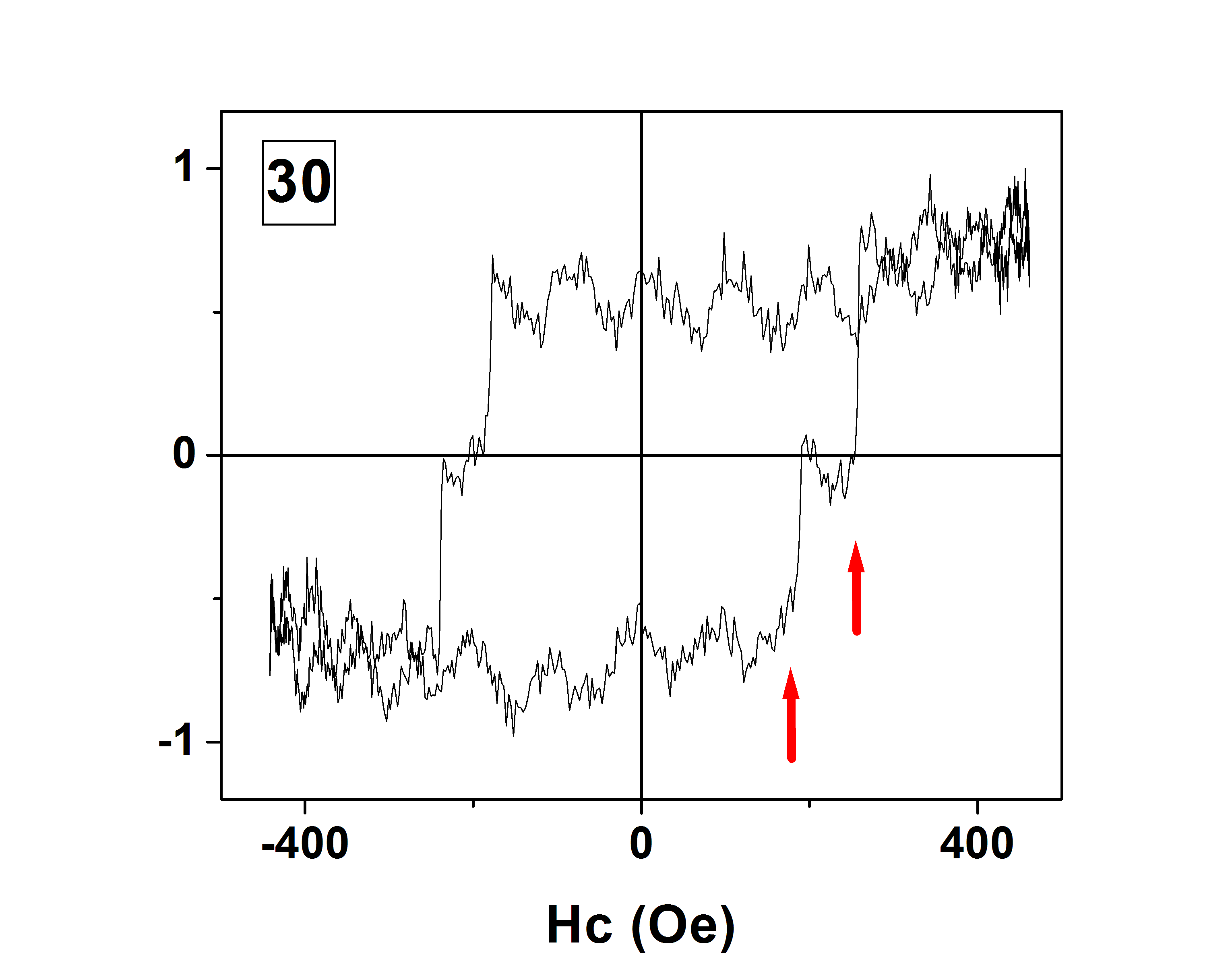 | 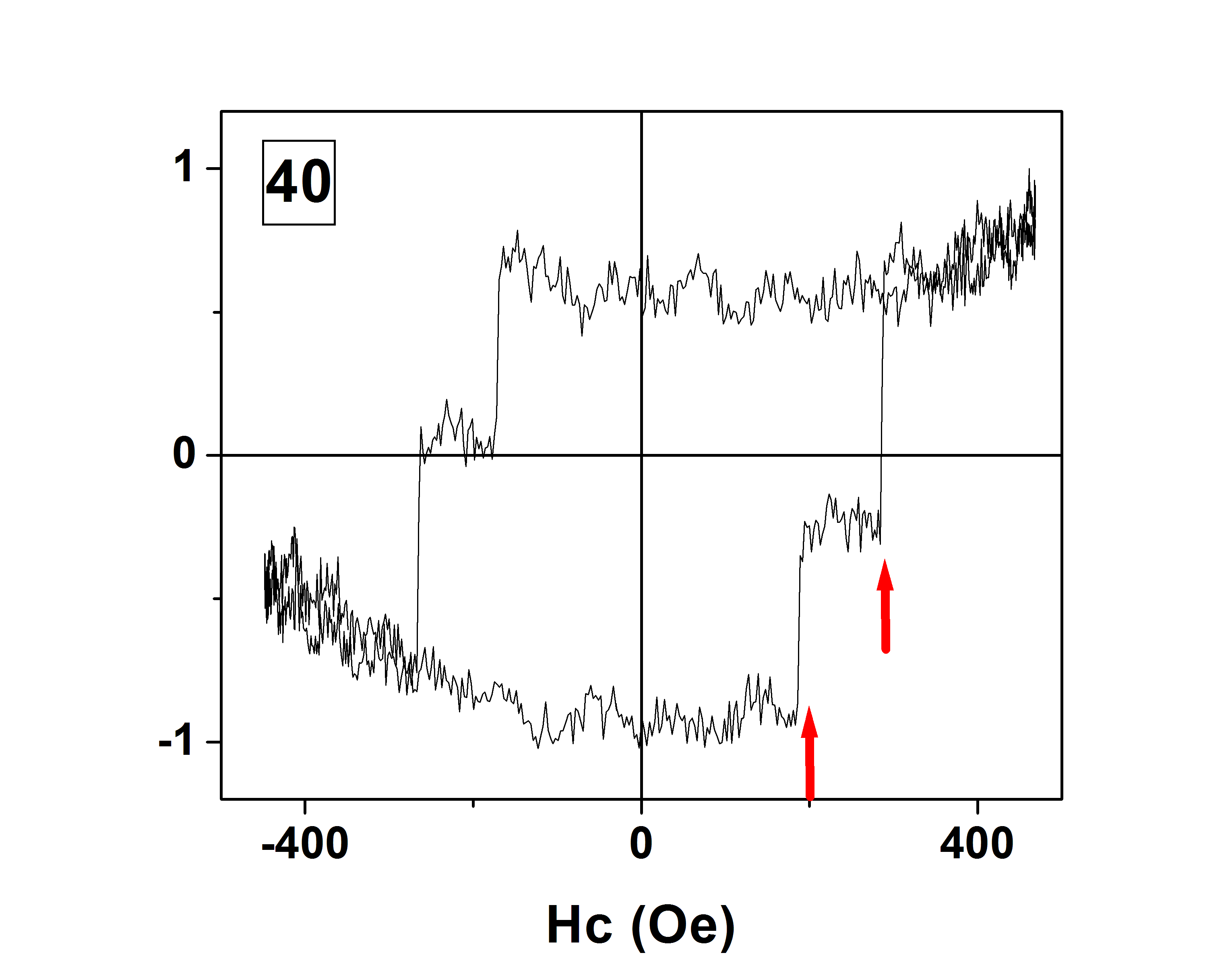 | 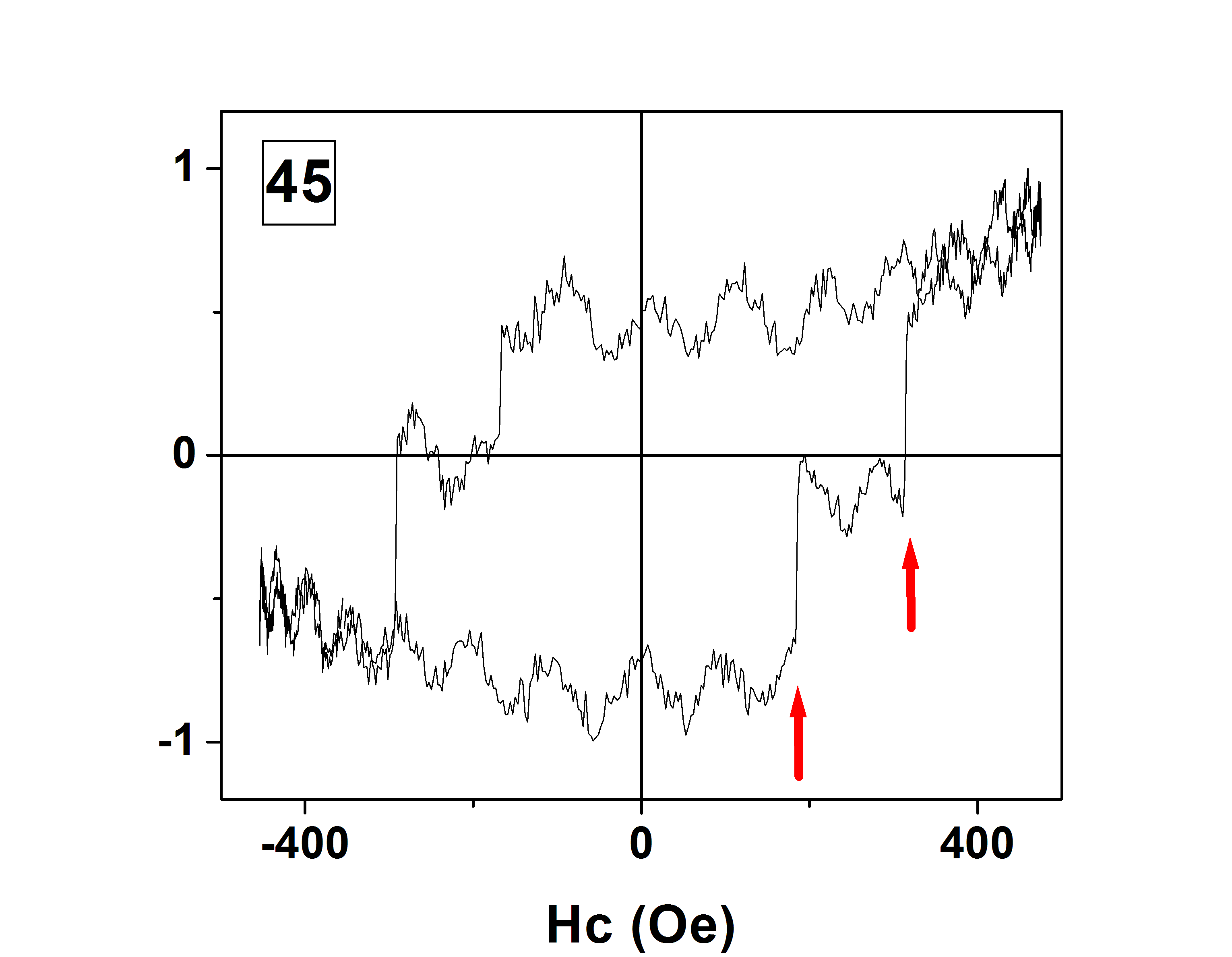 | 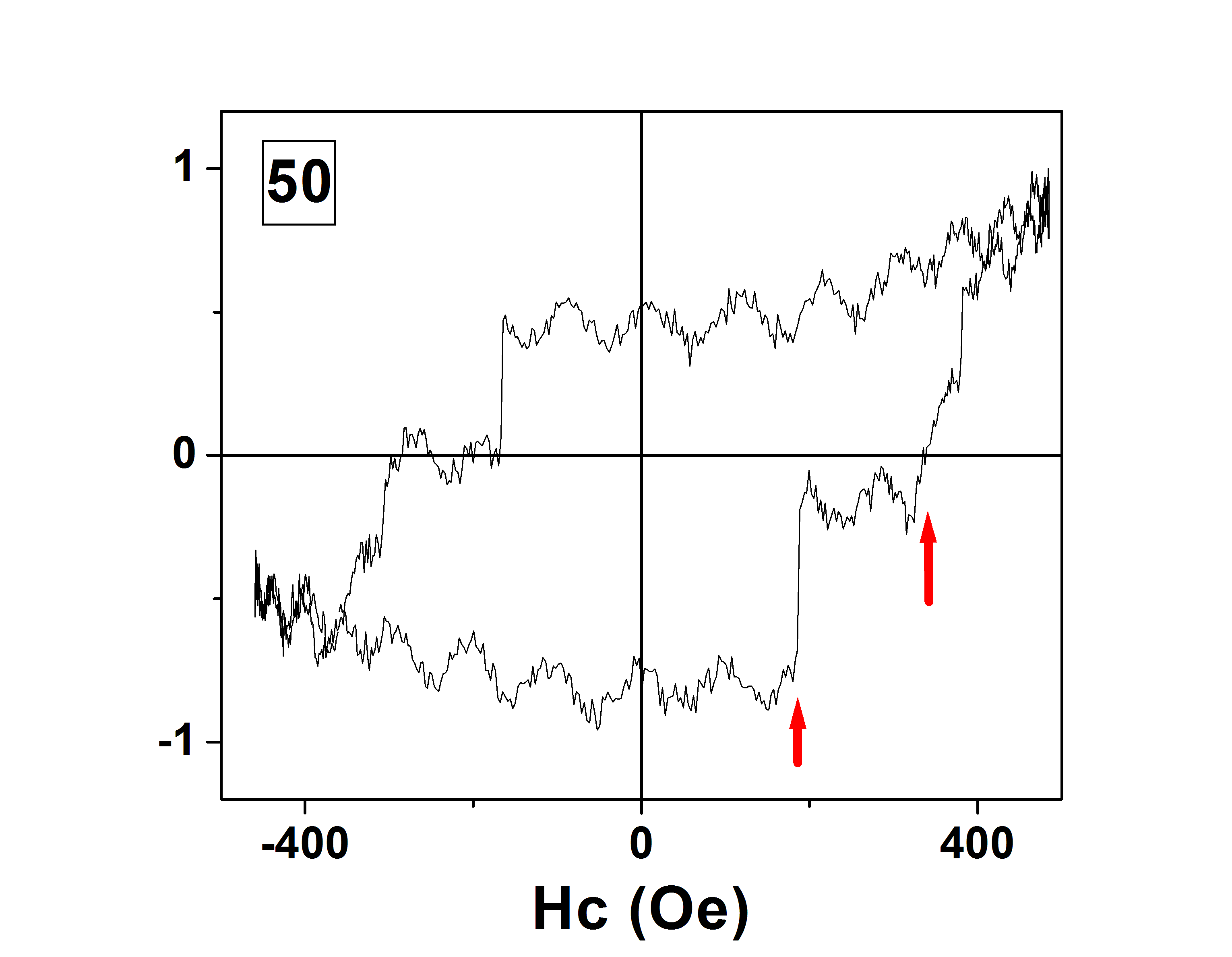 |
| **I-2** | 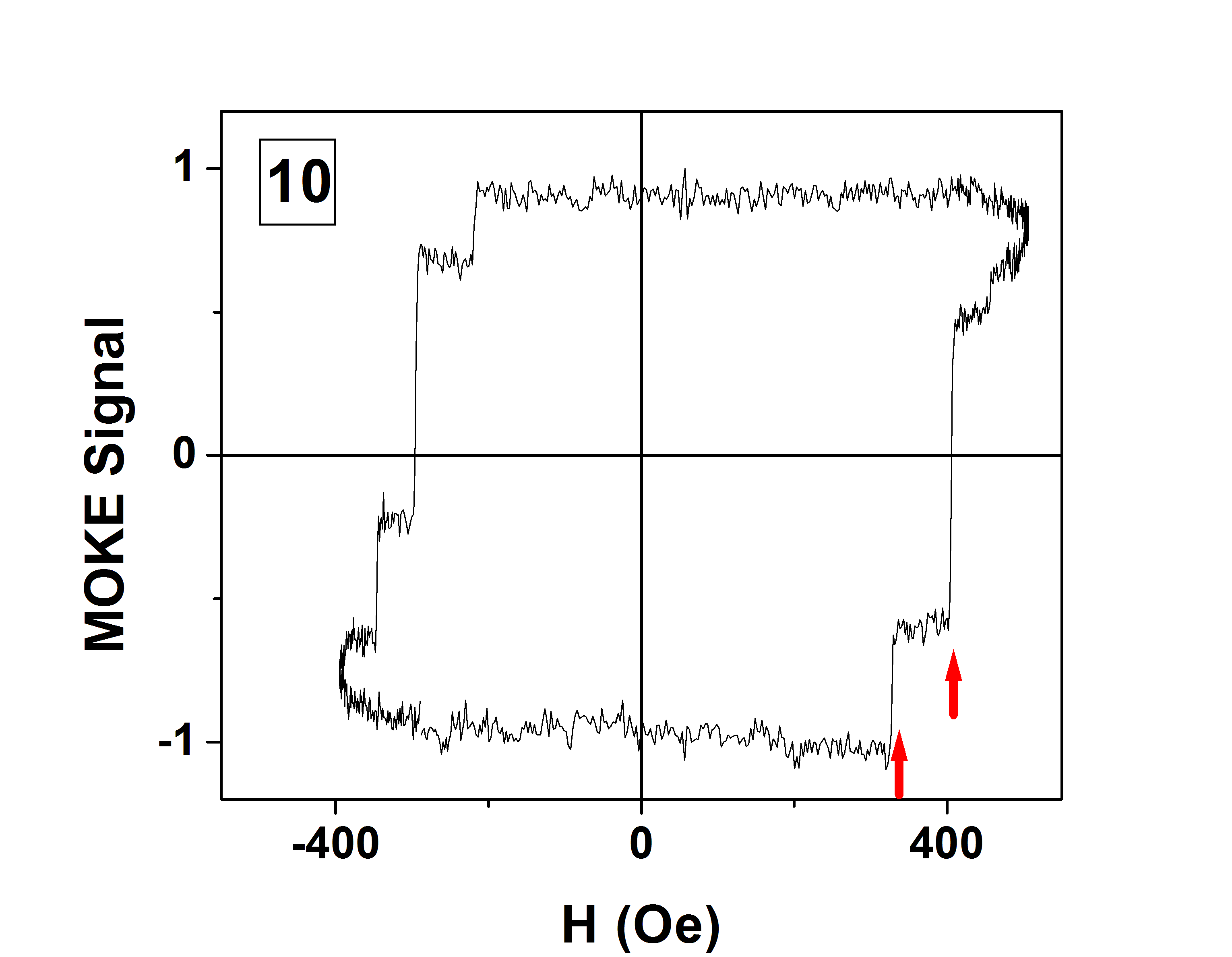 | 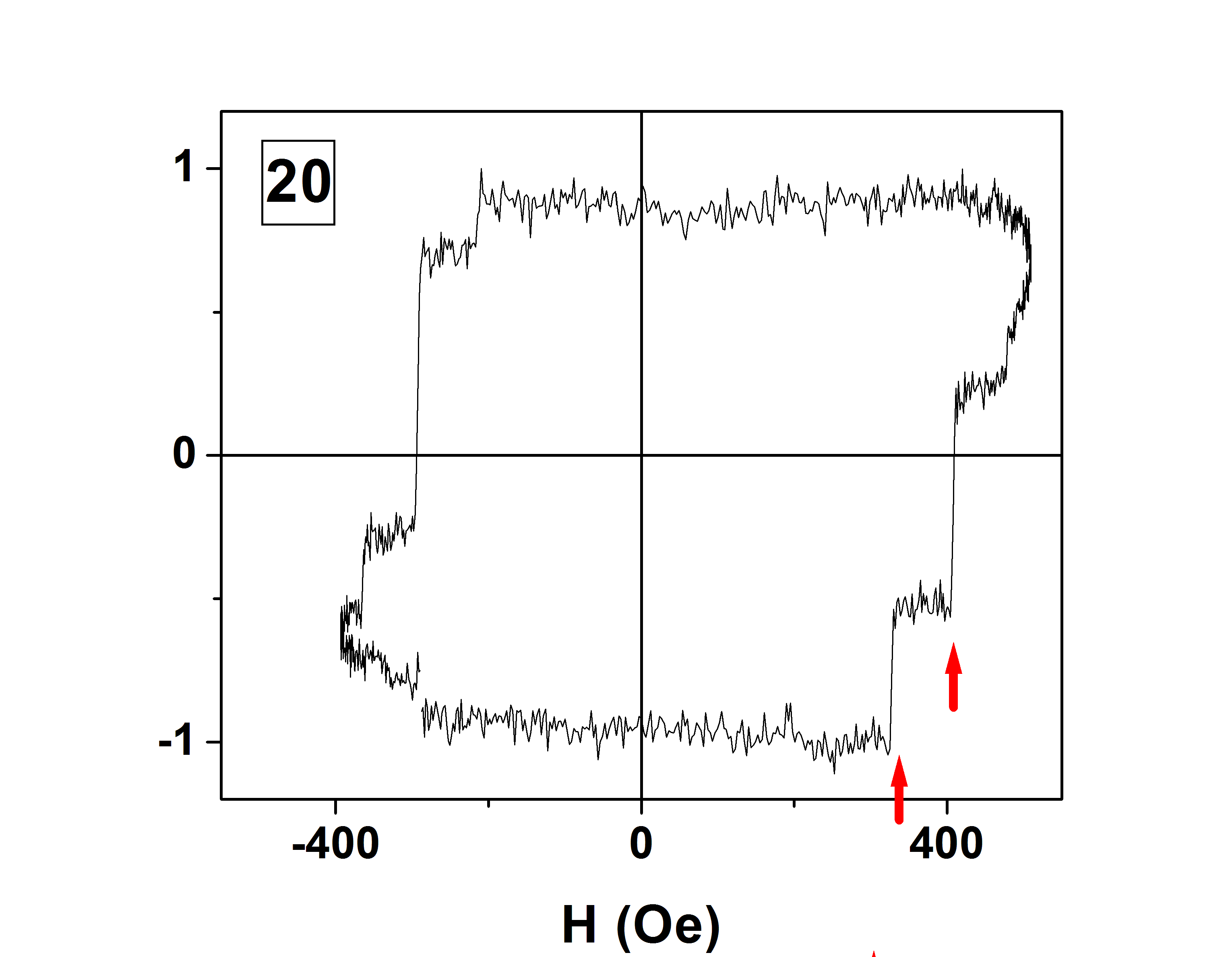 | 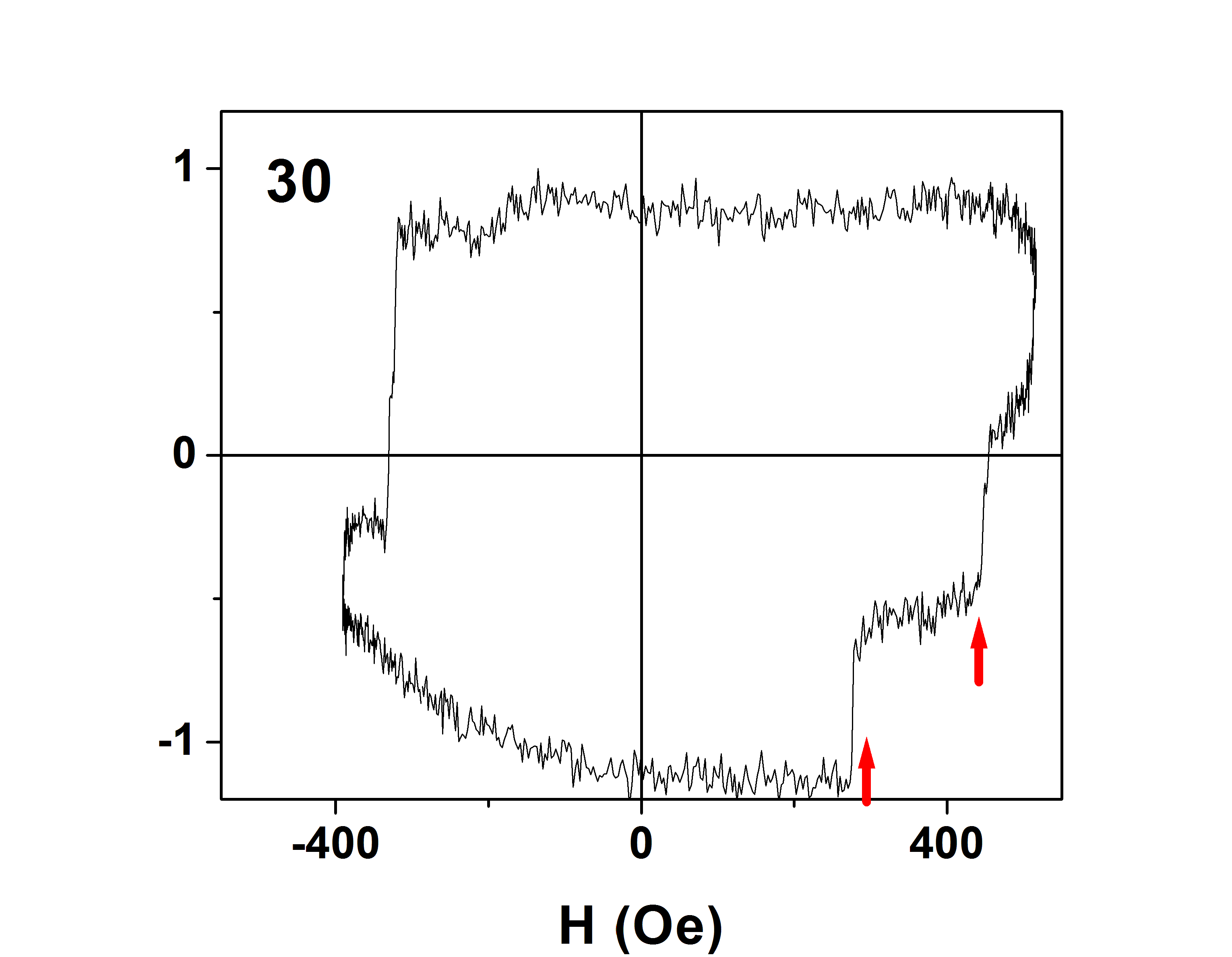 | 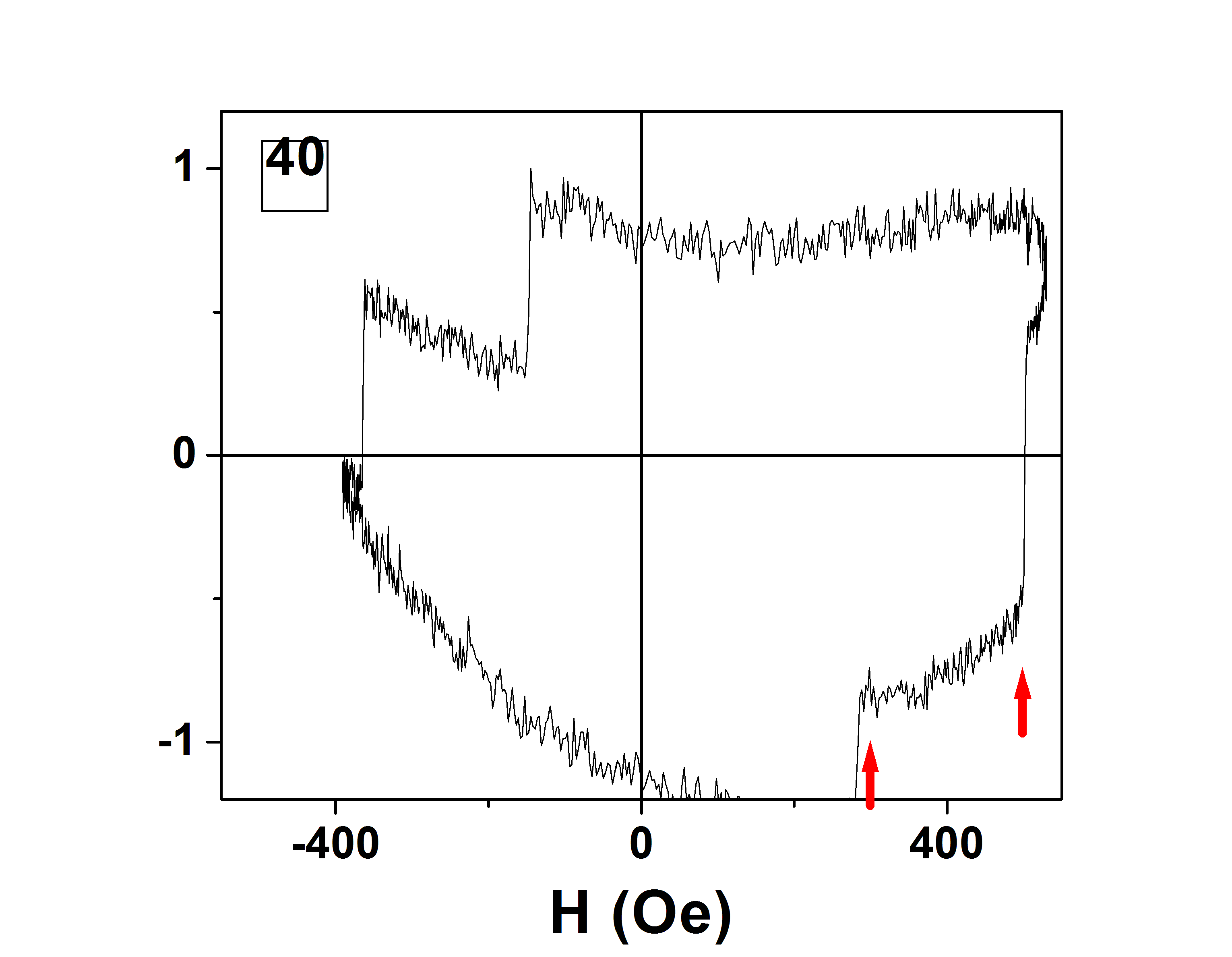 | 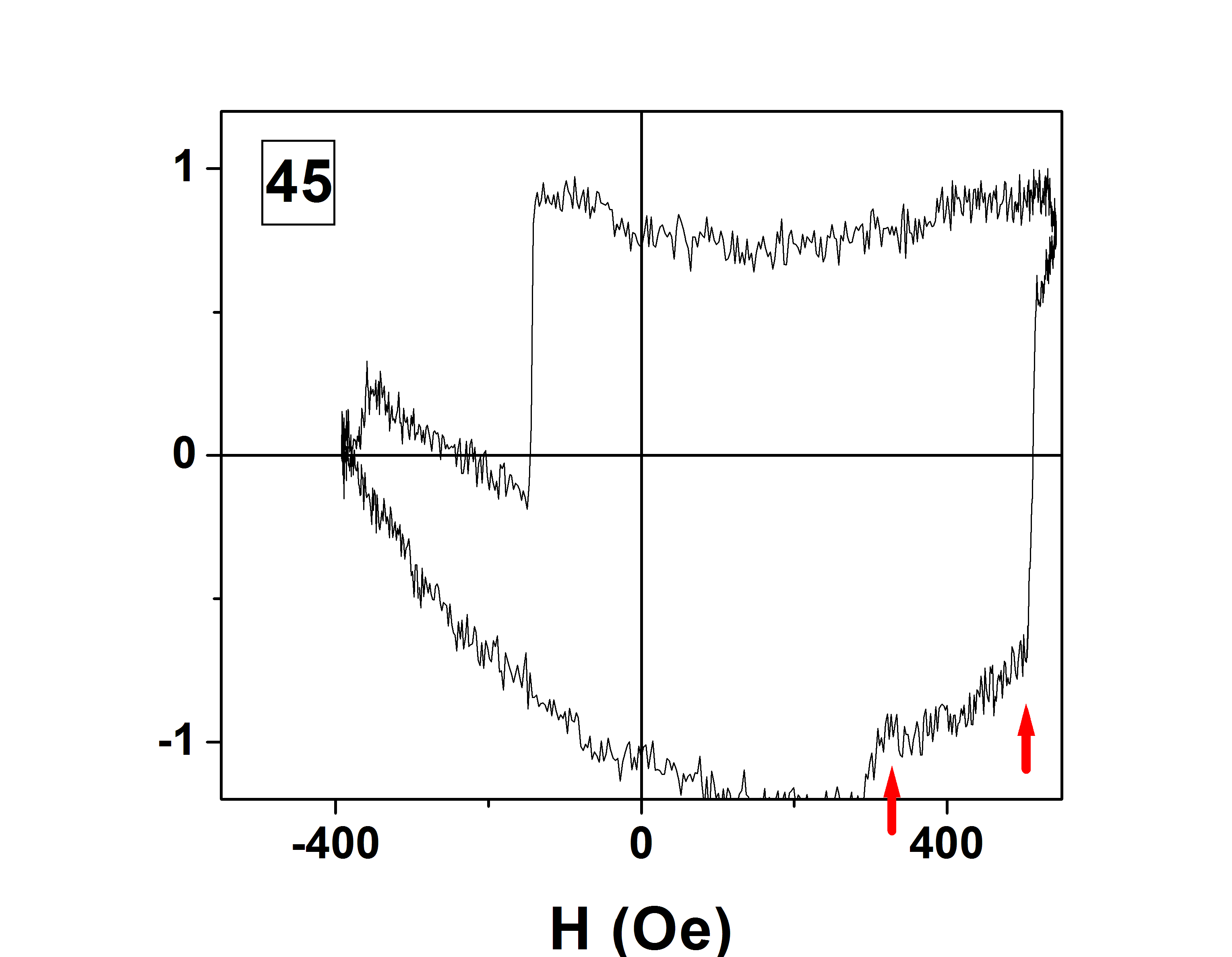 |  |
| **I-3** | 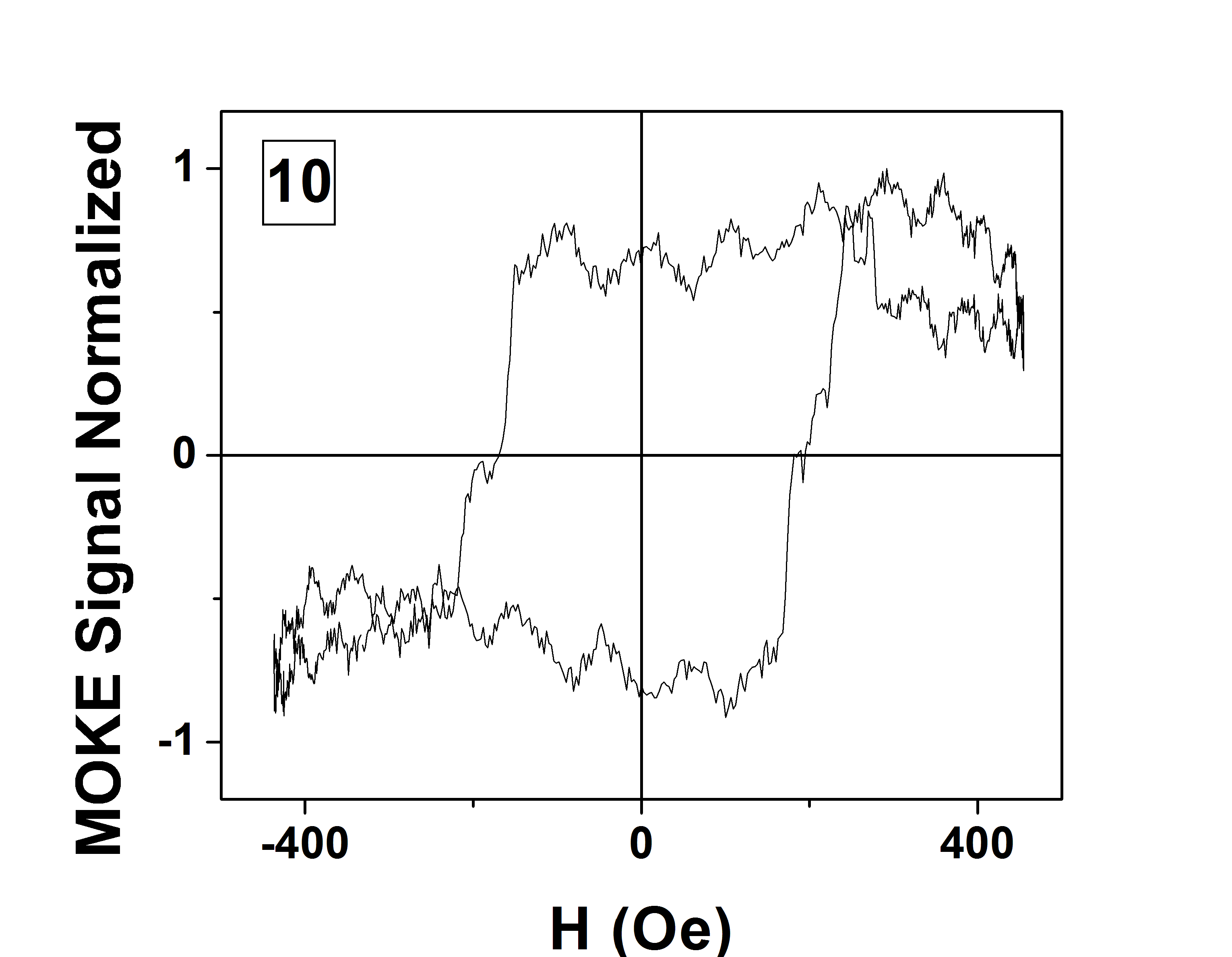 | 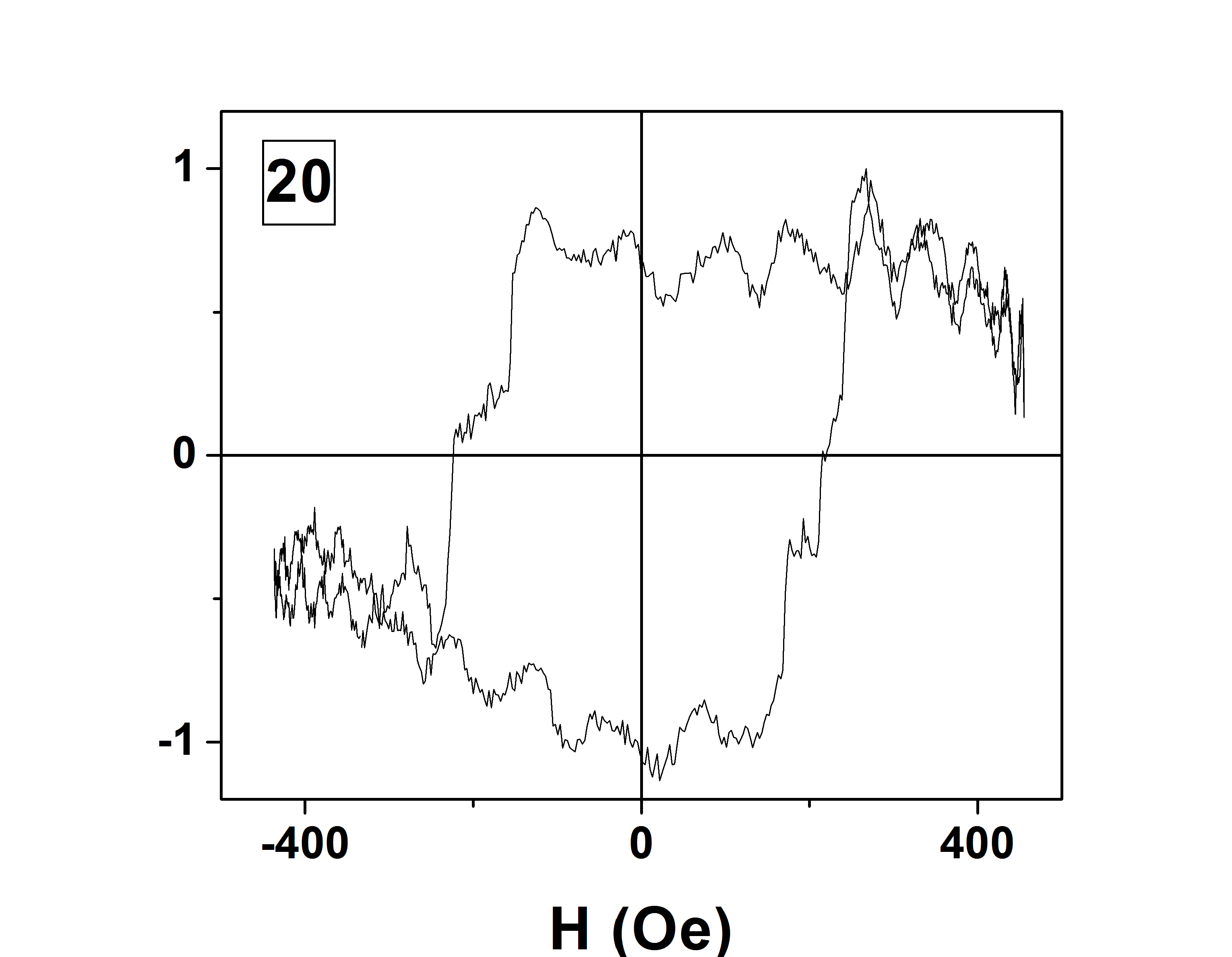 | 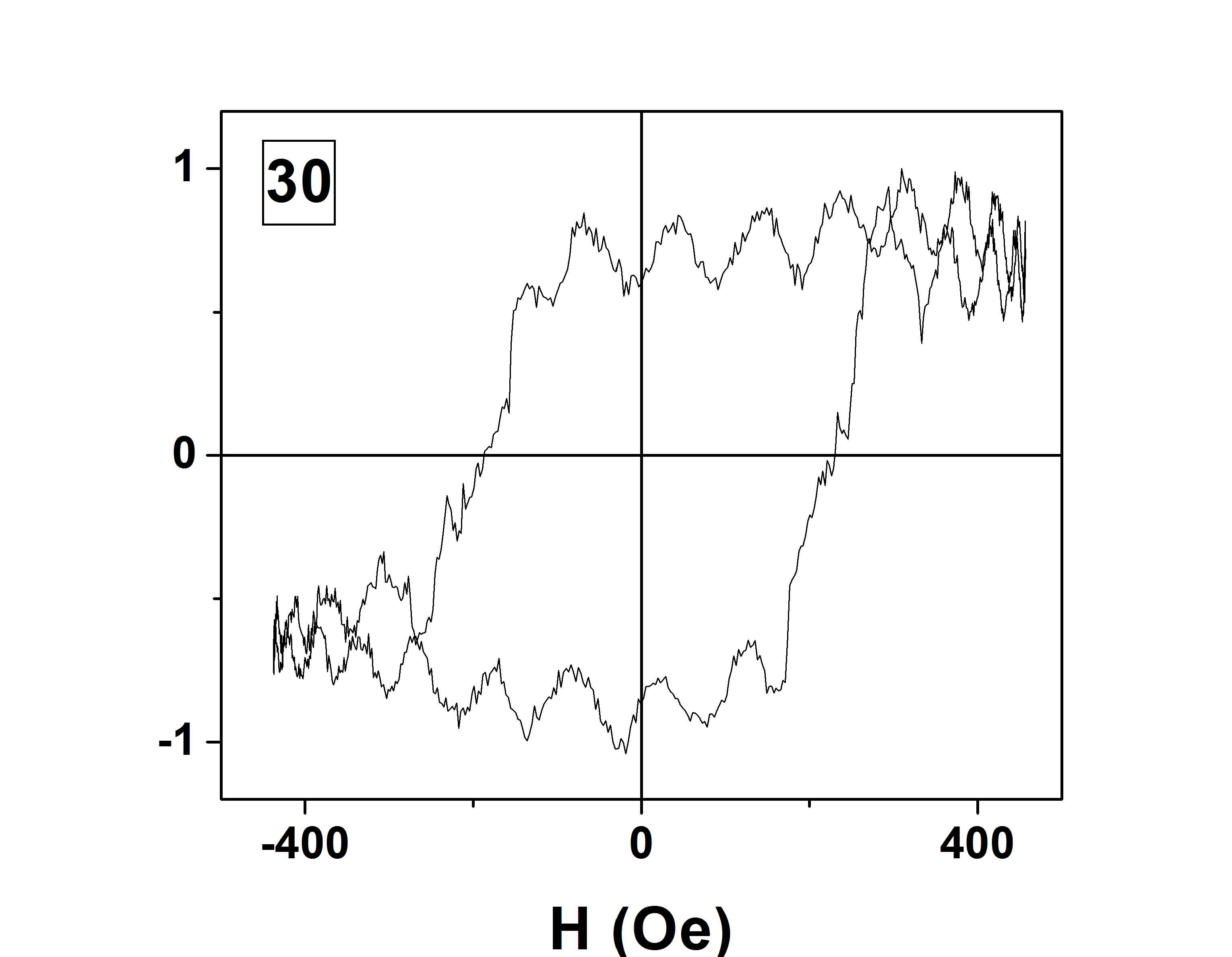 | 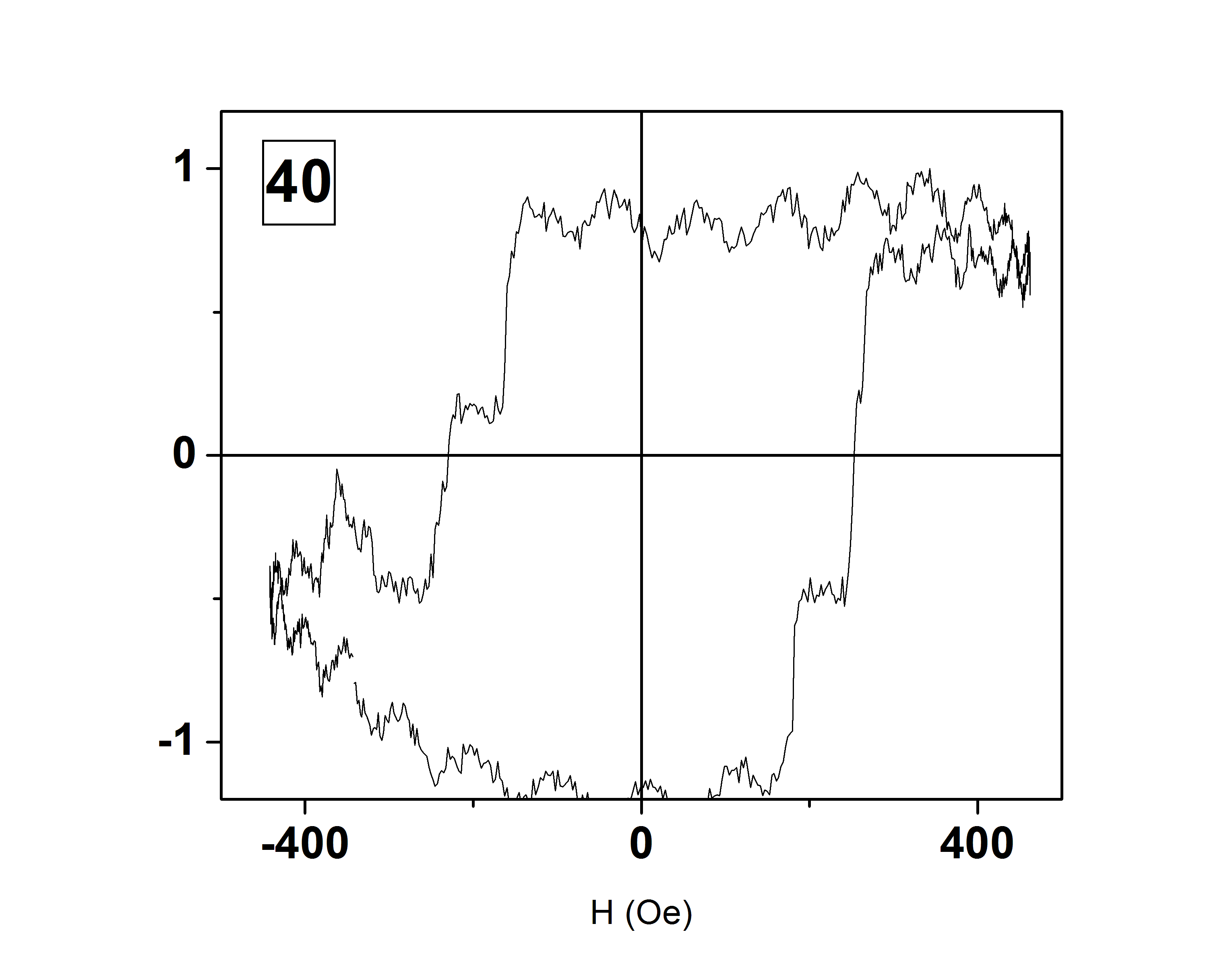 | 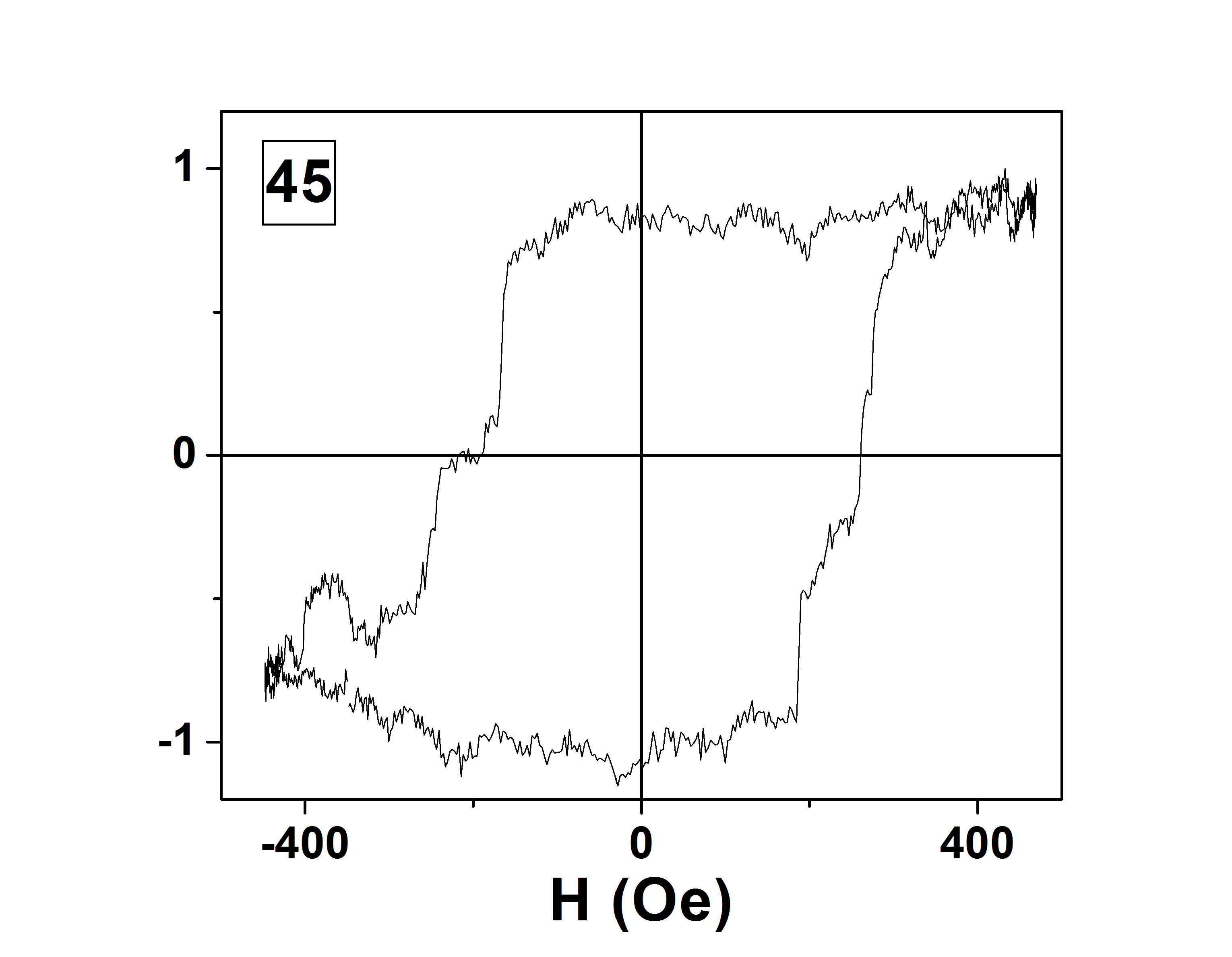 | 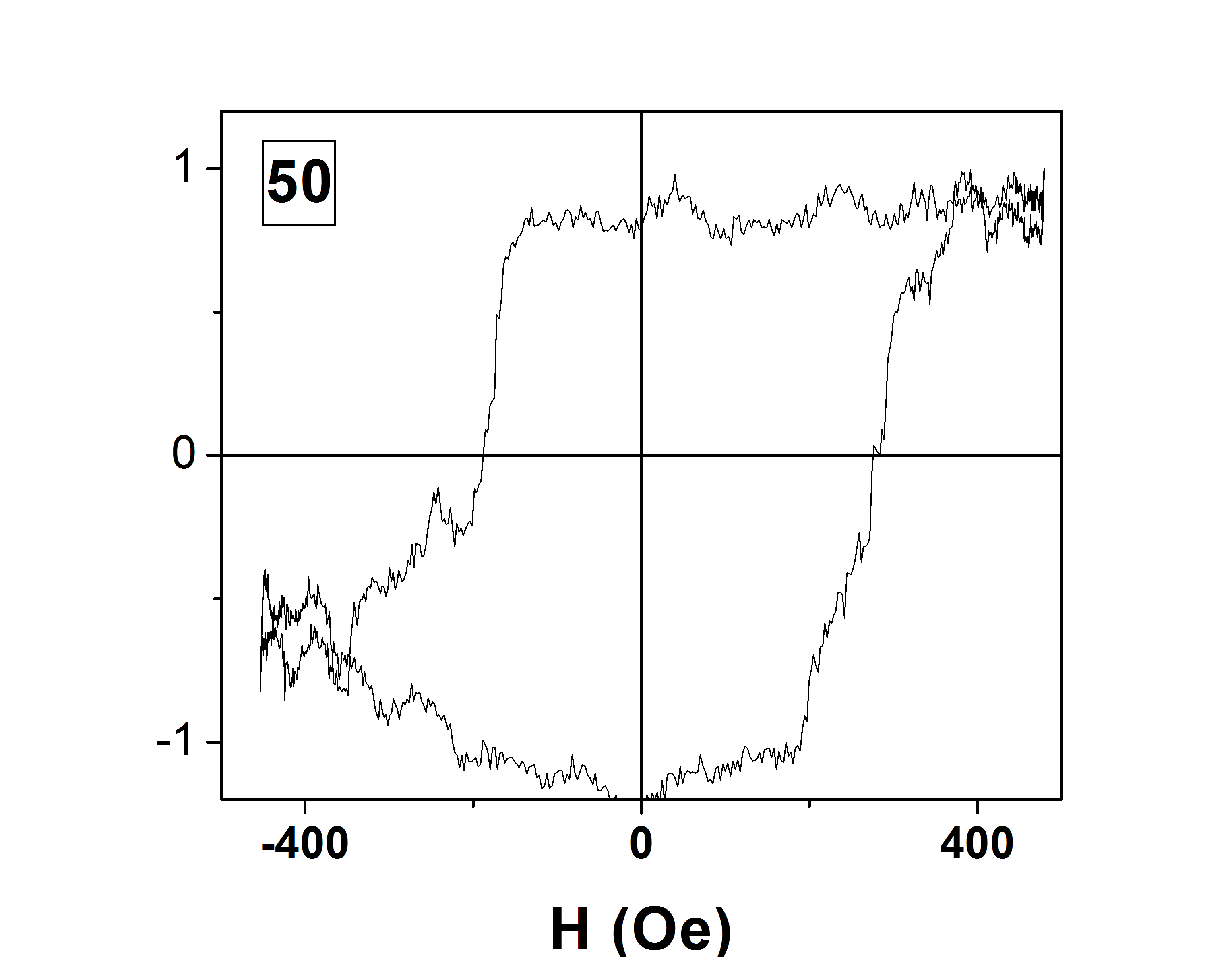 |
| **II-5** | 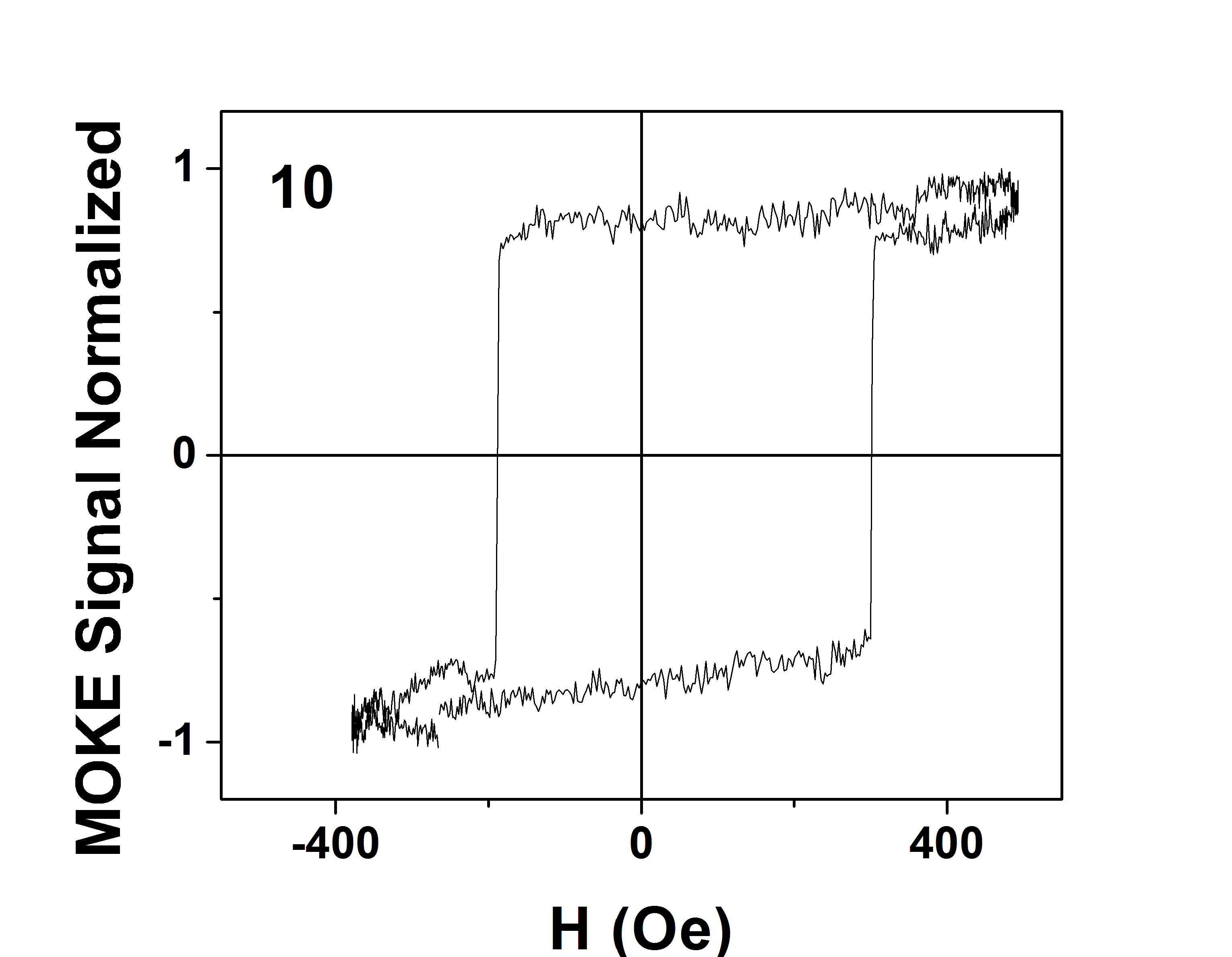 | 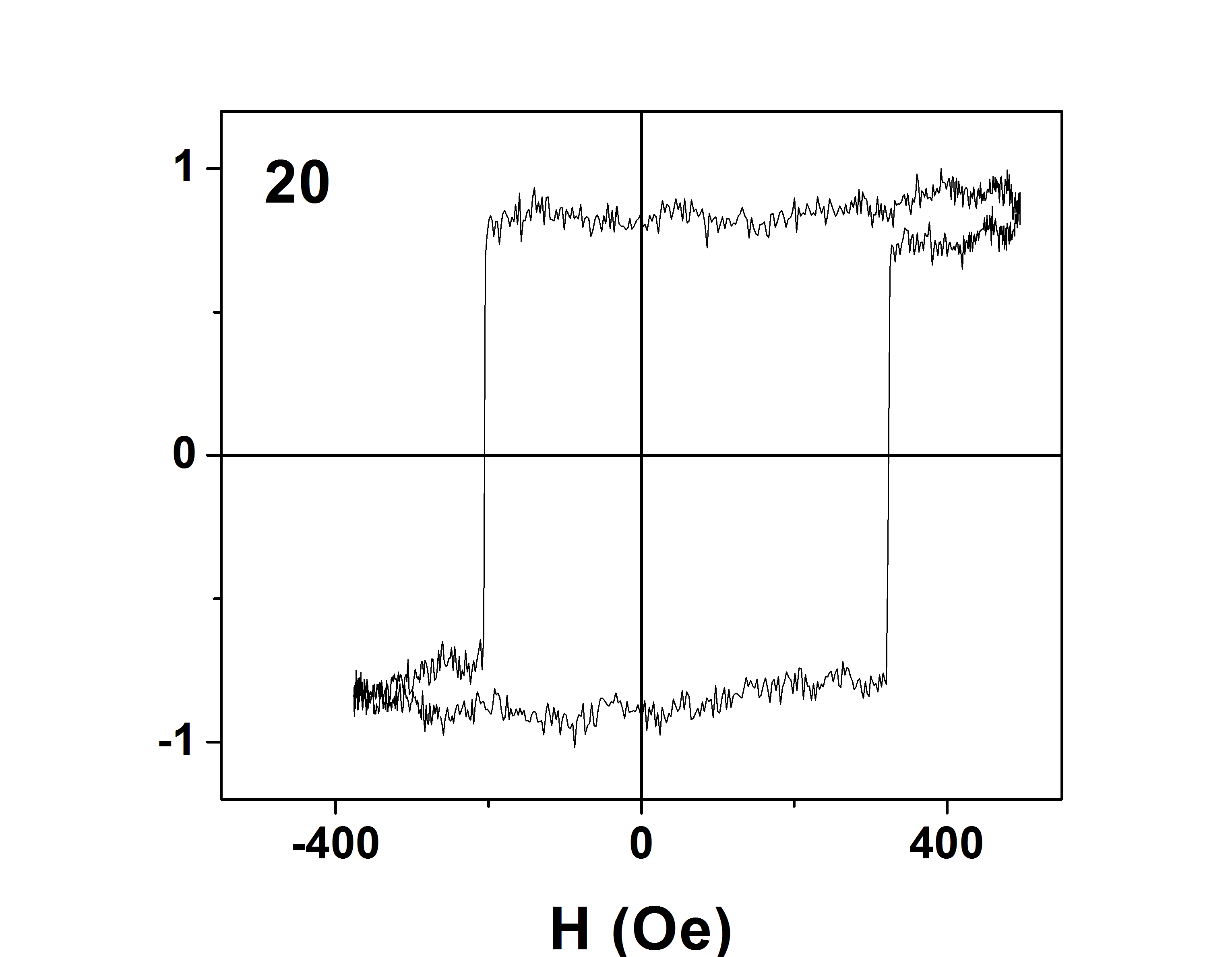 | 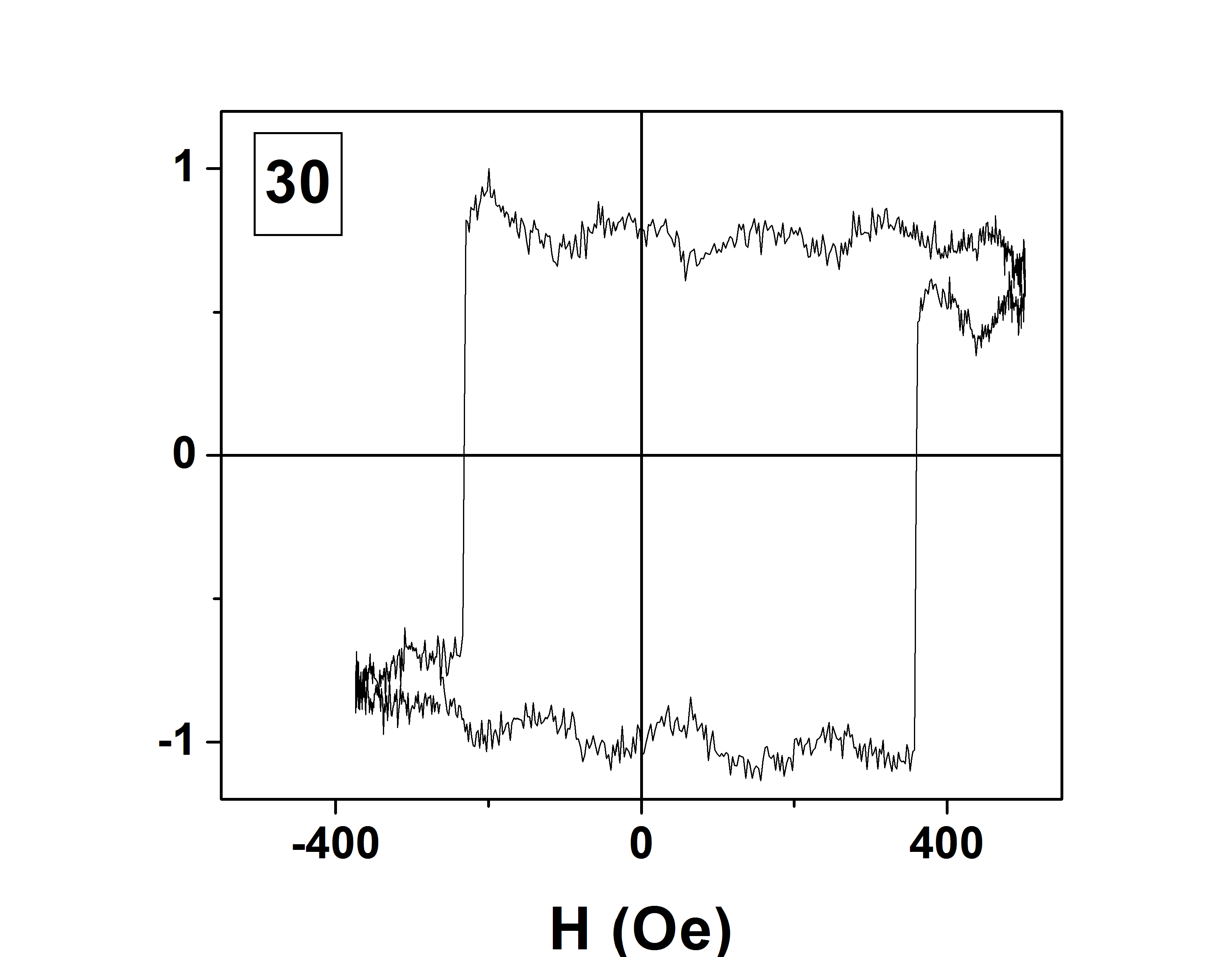 | 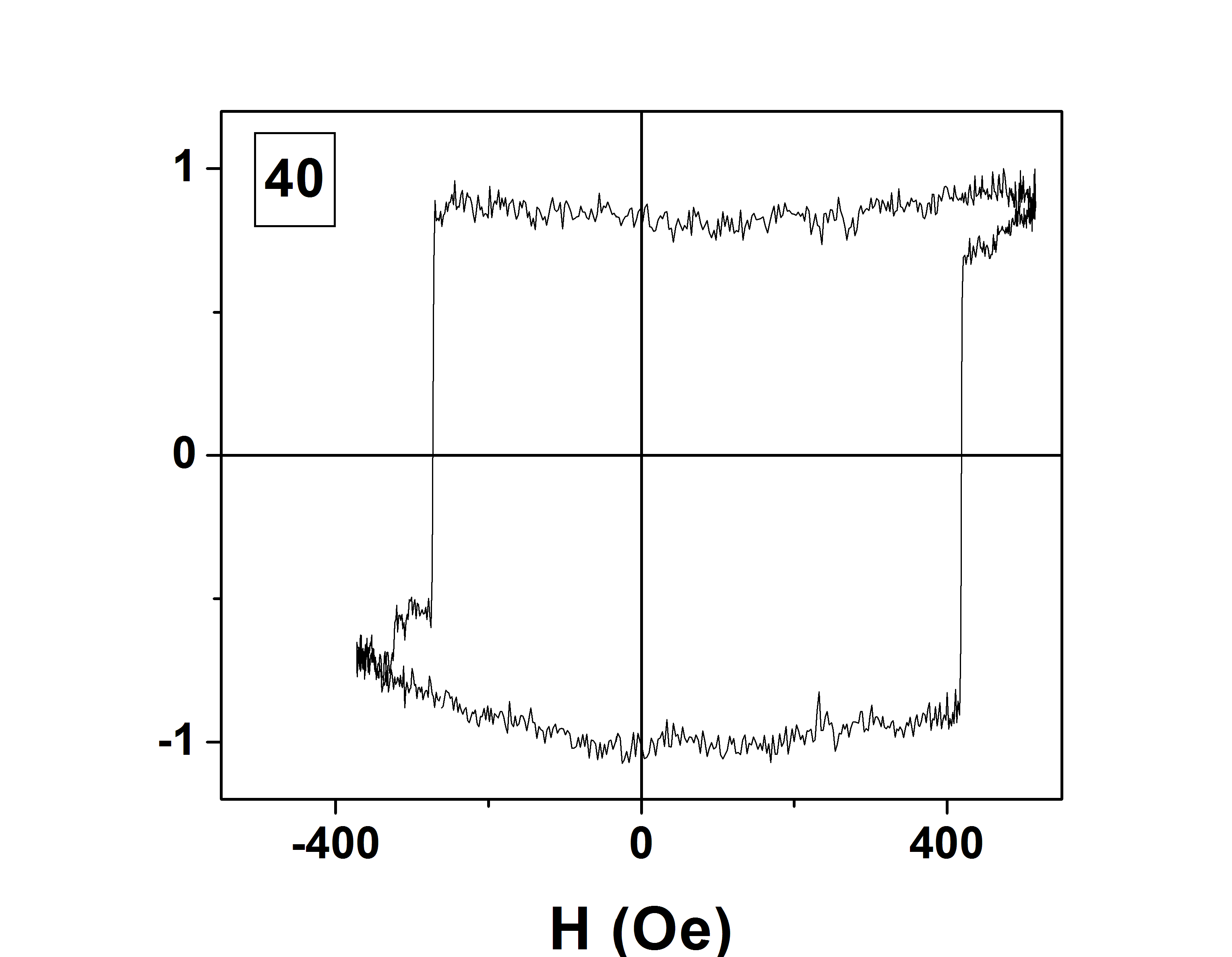 | 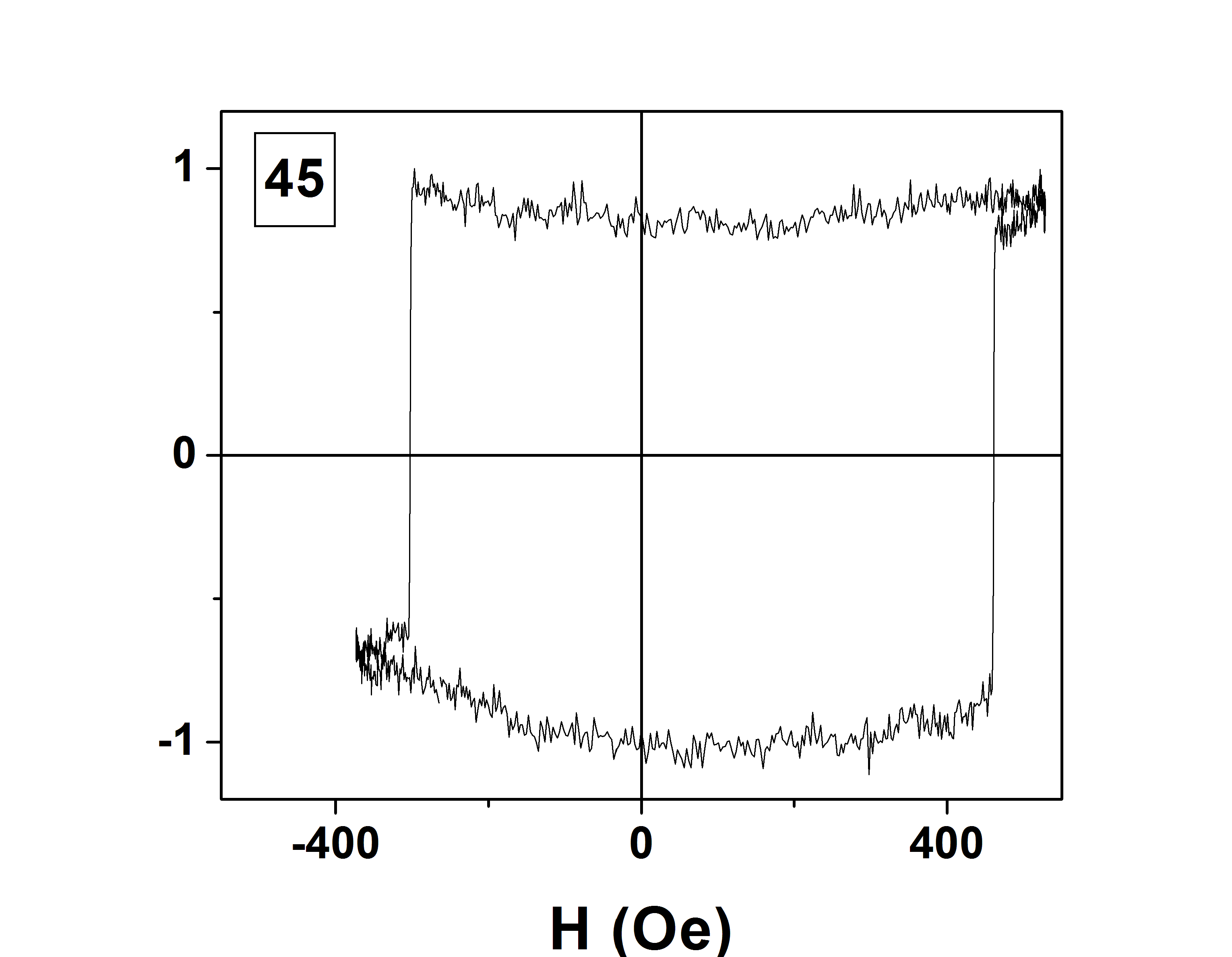 | 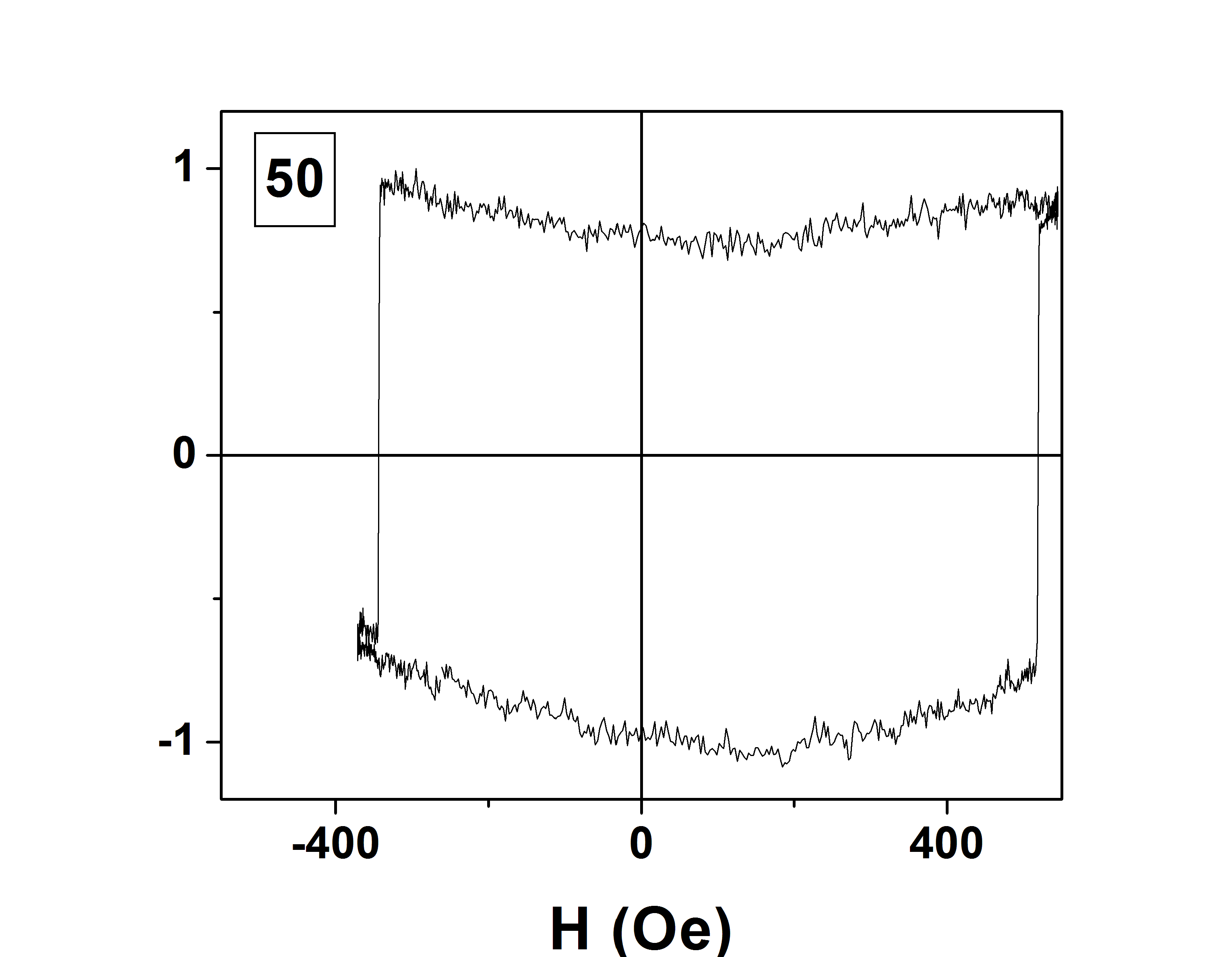 |
| **II-6** | 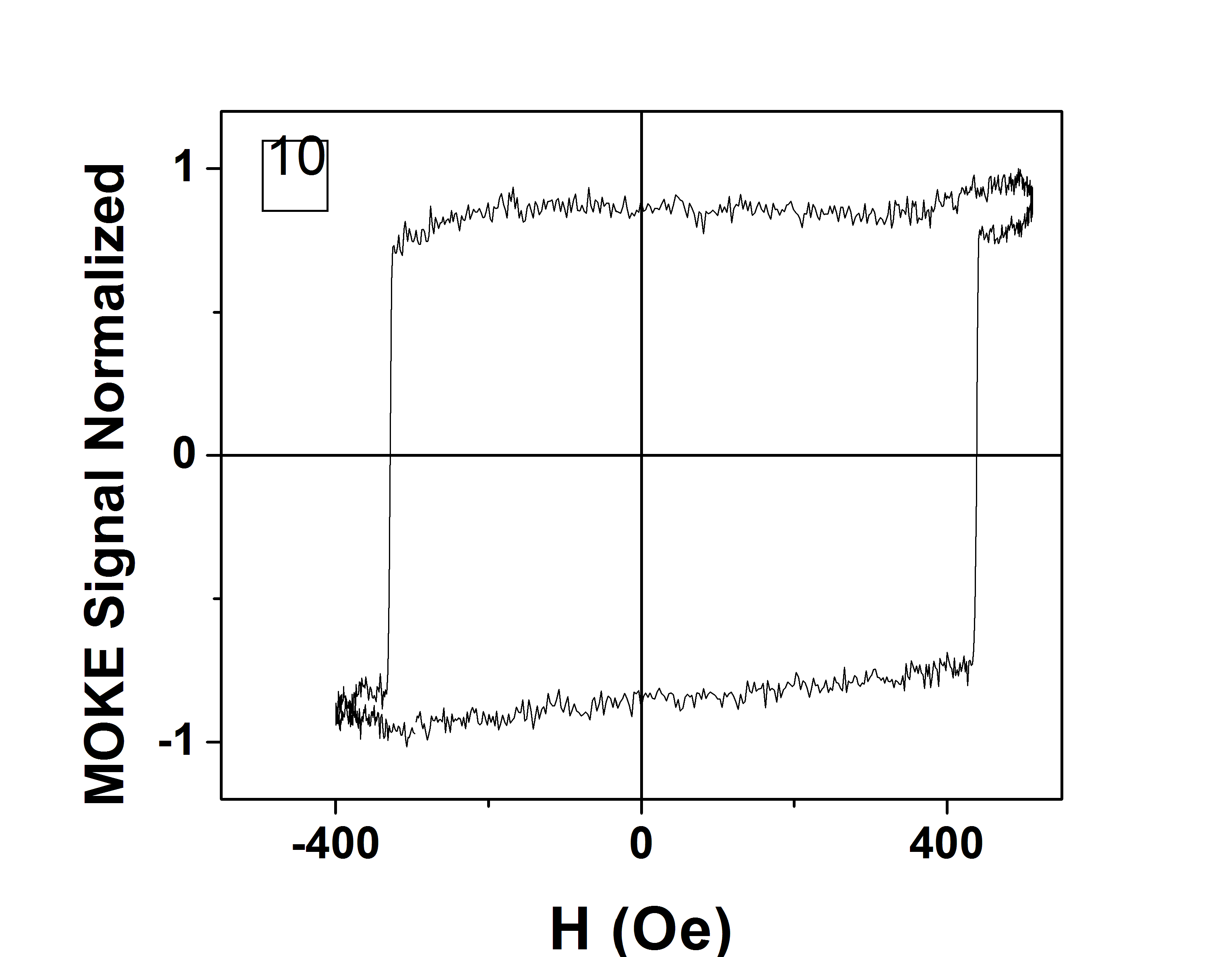 | 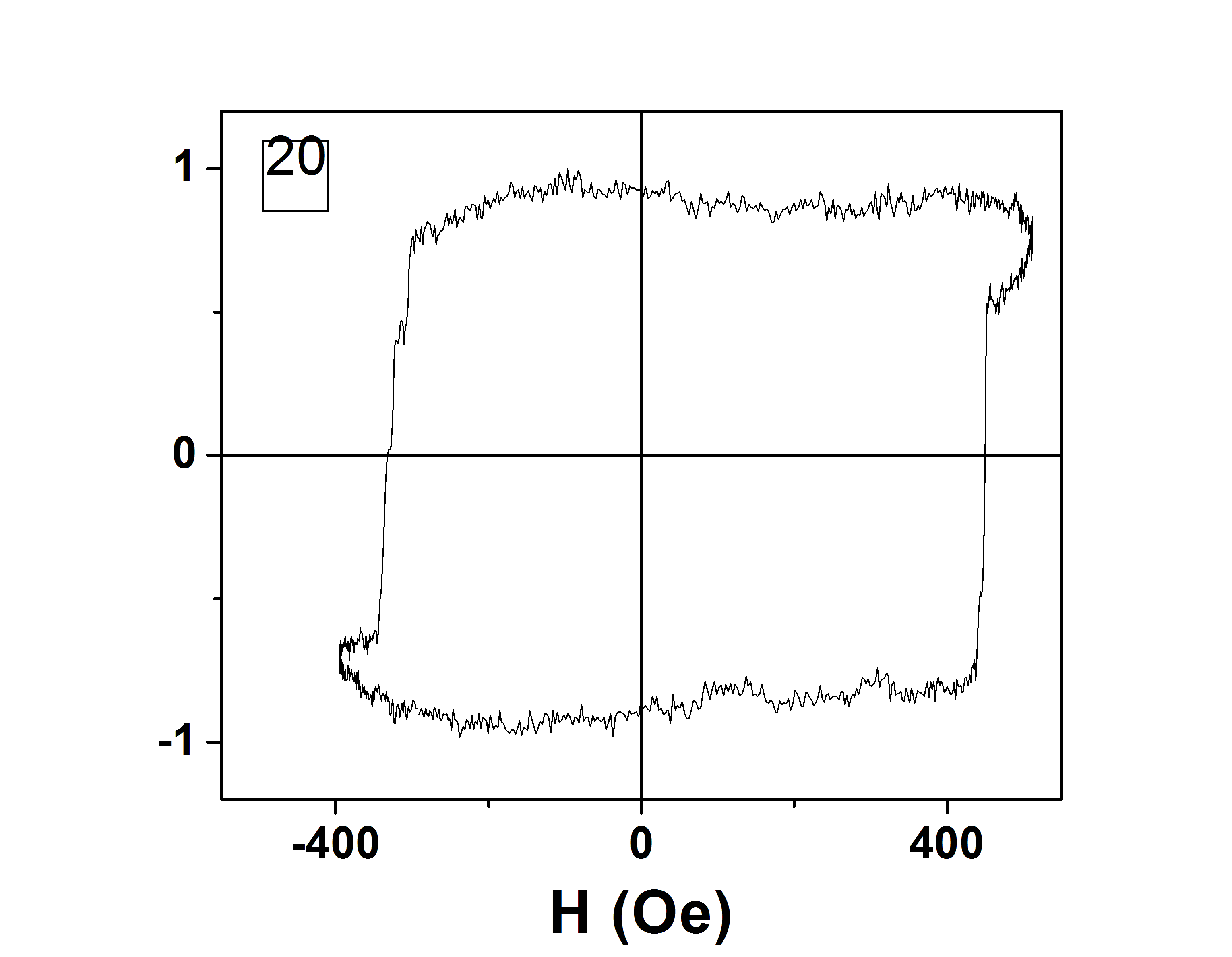 | 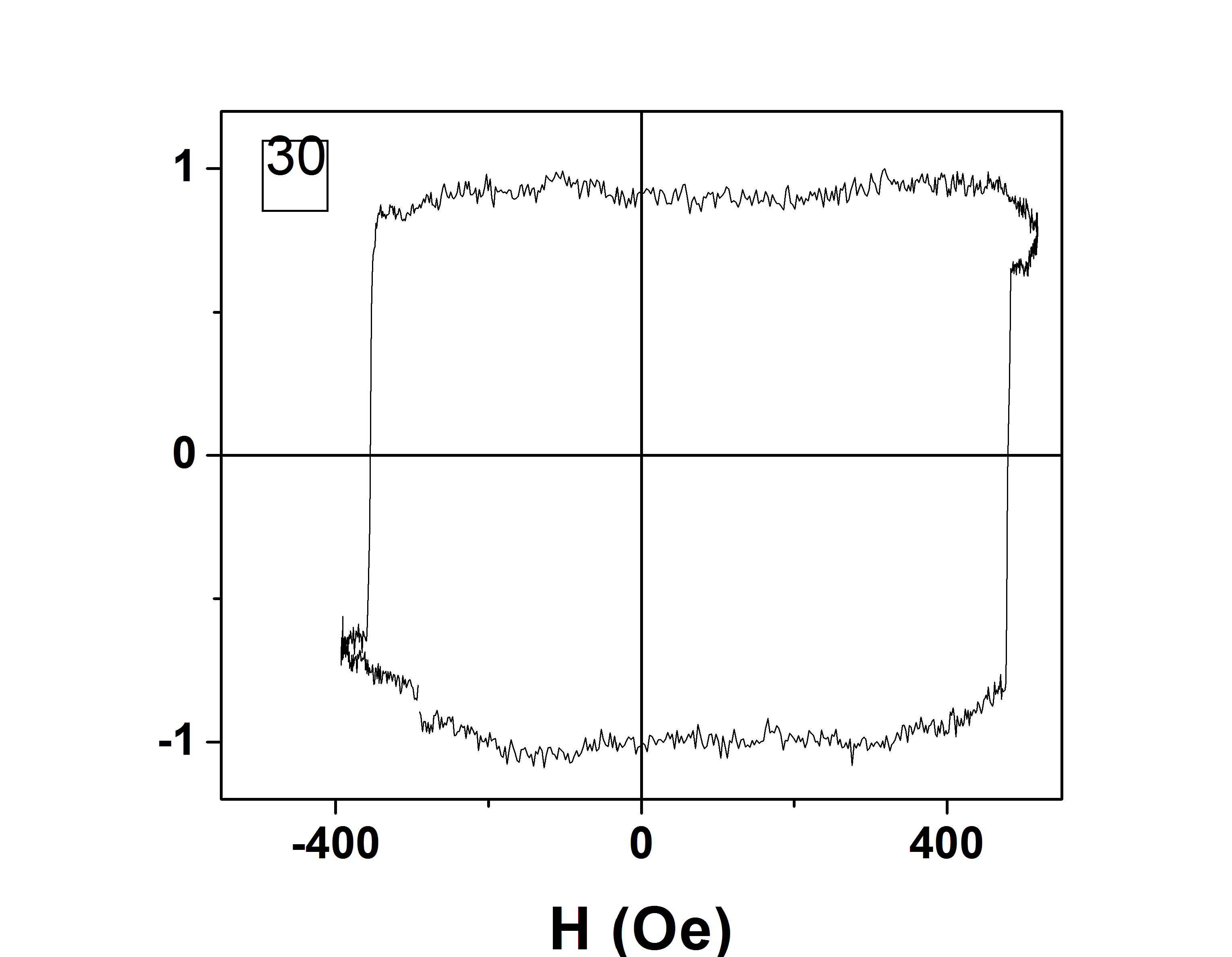 | 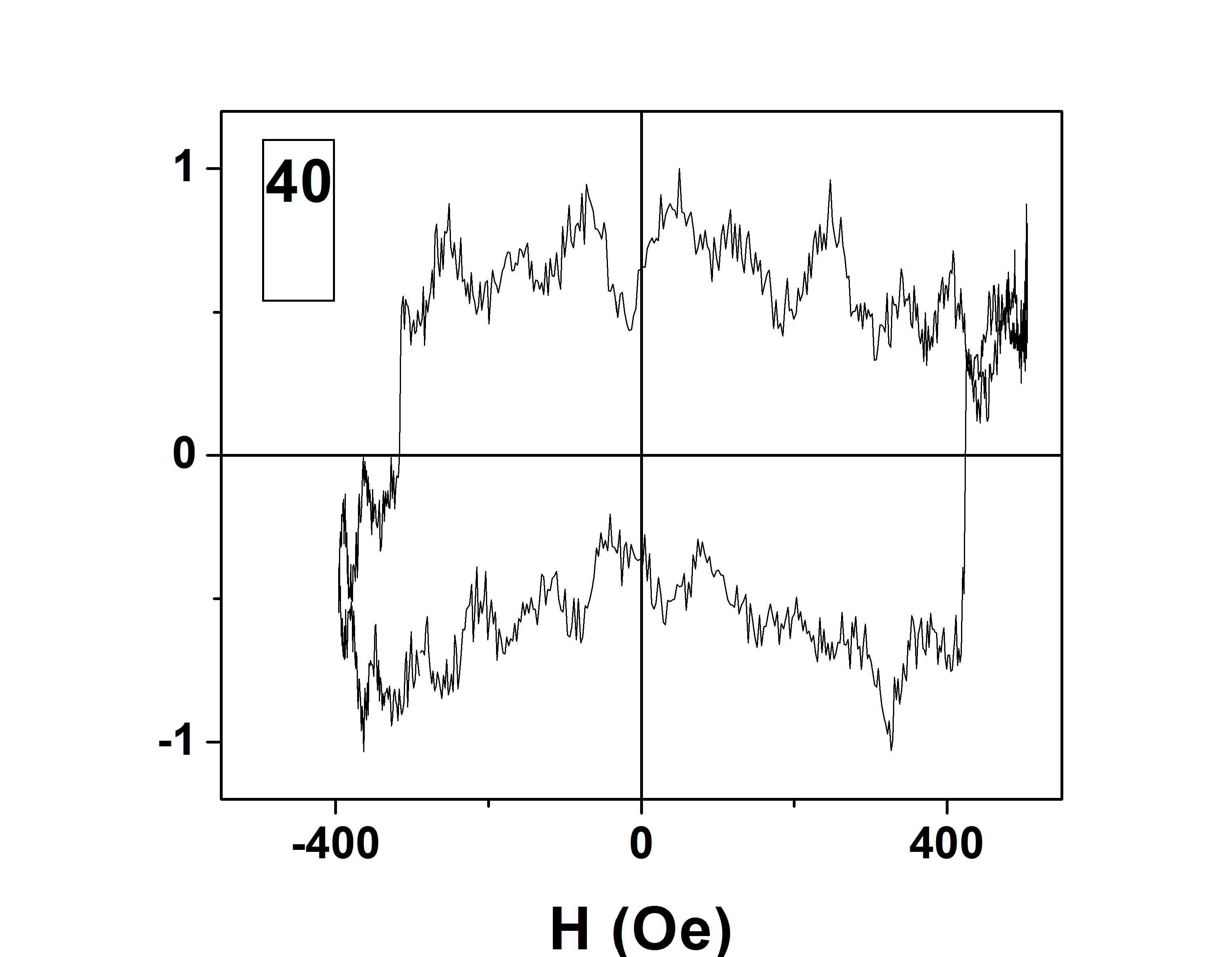 | 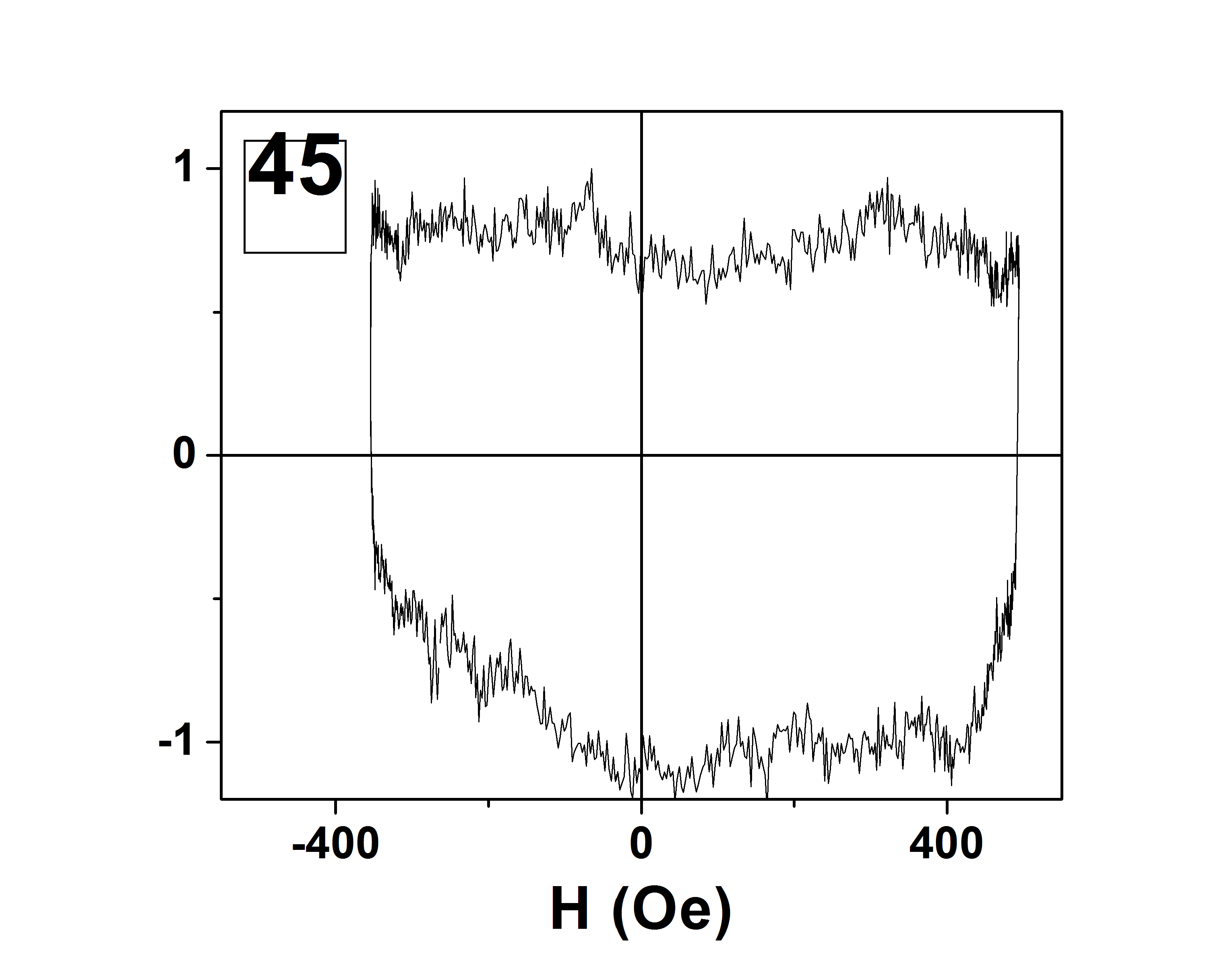 | 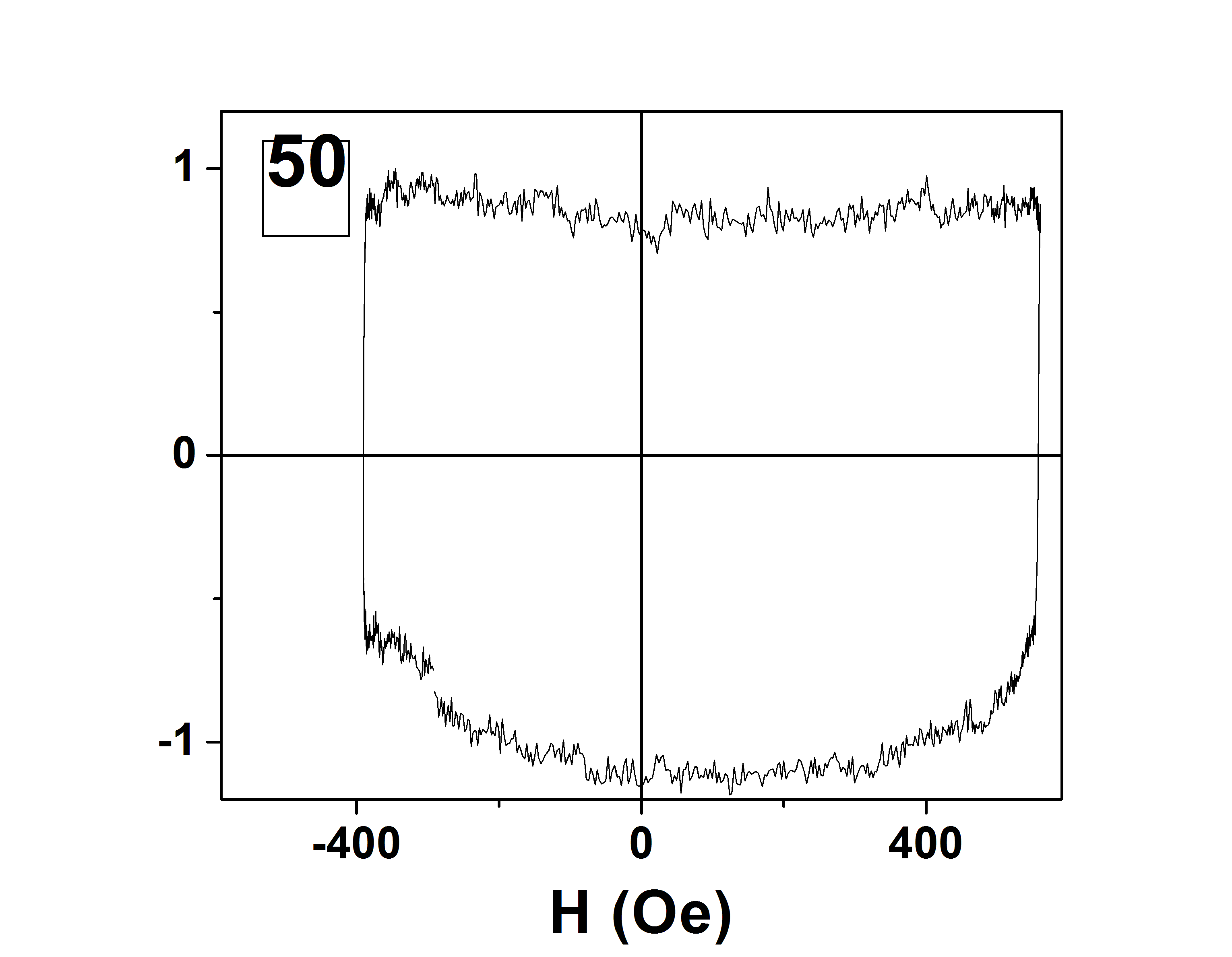 |


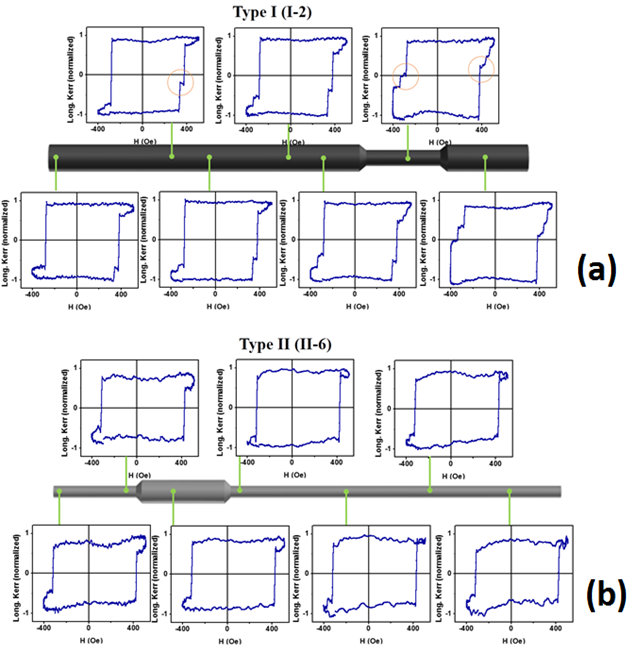


**Figure 2S** Positionally tracked MOKE hysteresis loops measured along individual nanowires of type I (top panel, I-2 NWs) and type II (bottom panel, II-6 NWs). Schematic picture of each type of nanowires are shown.

**Numerical Simulations**


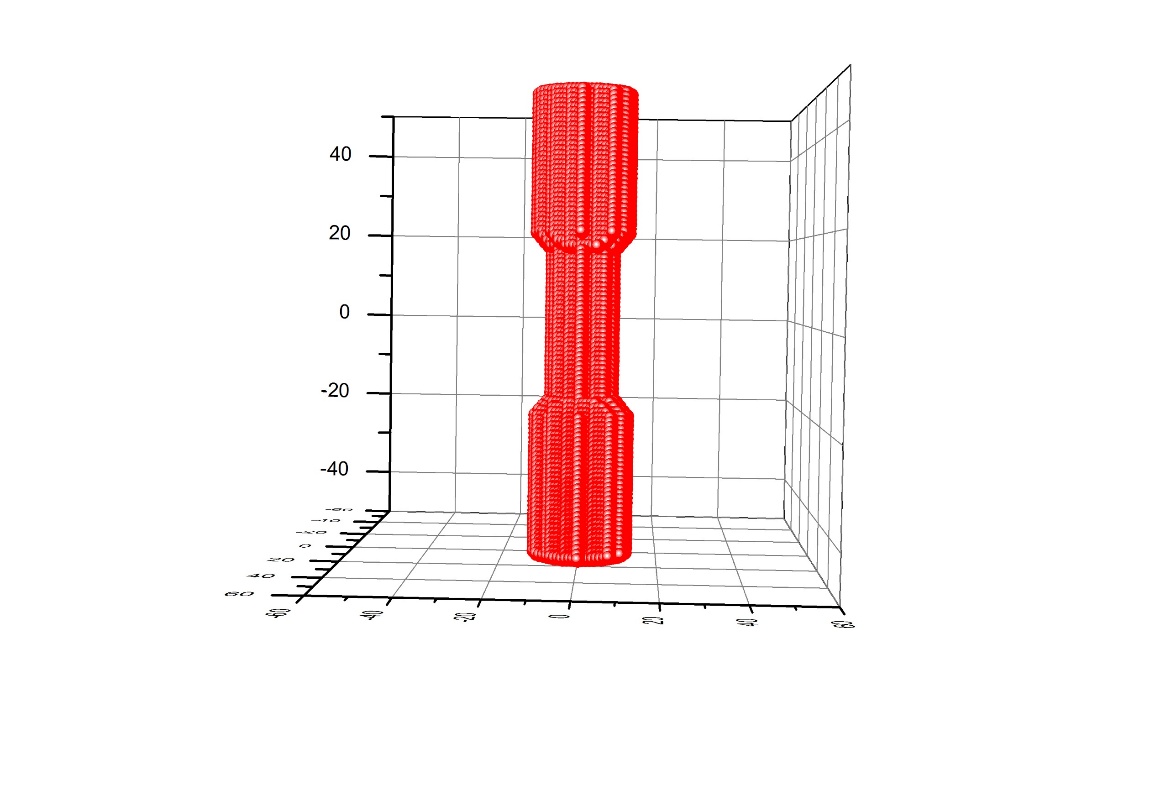
**(a)**


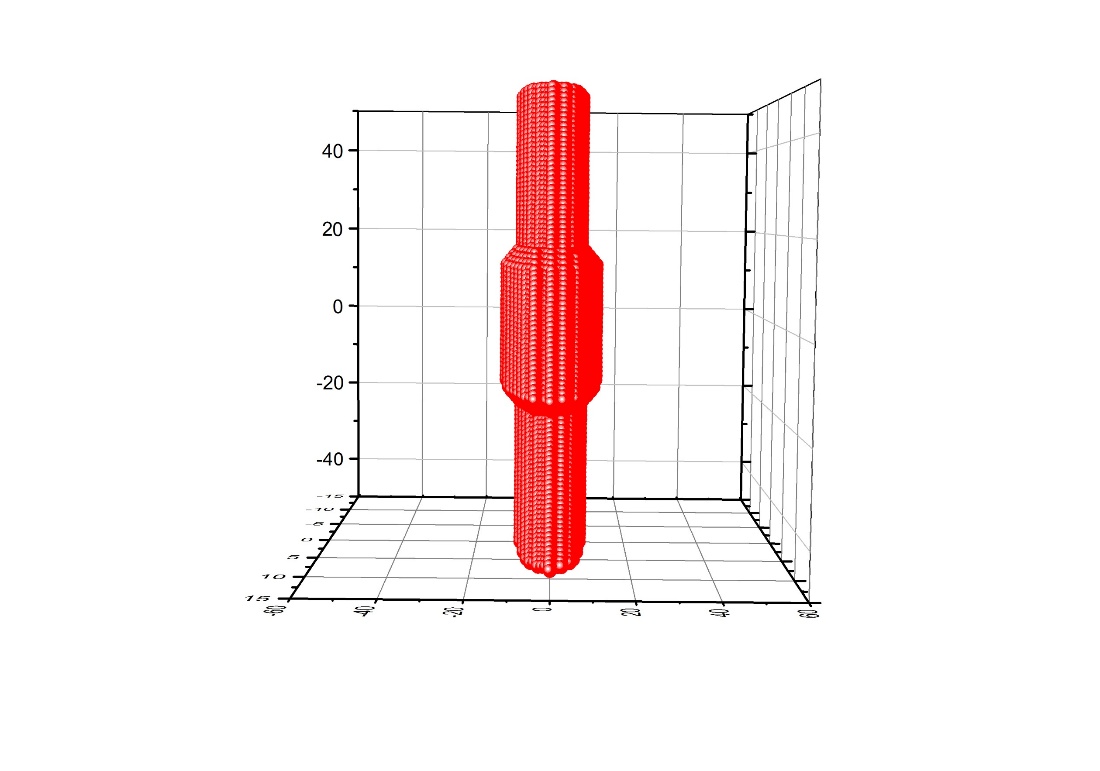
**(b)**


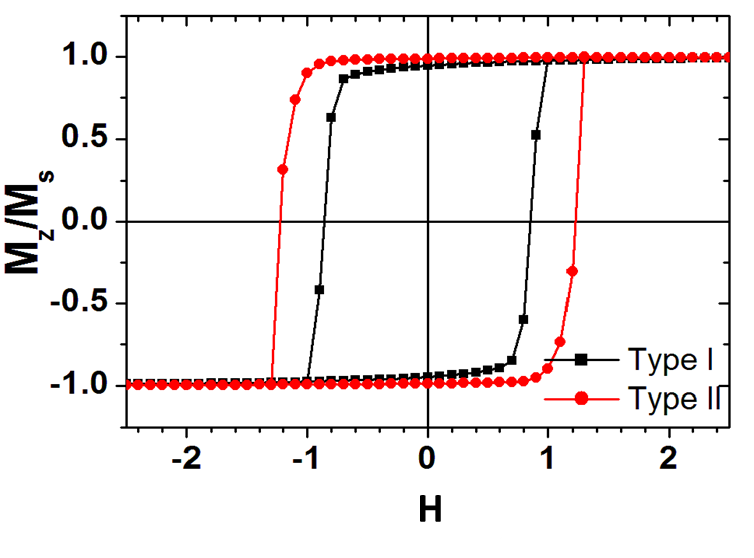
**(c)**

**Figure 3S.** Type-I (a) and type-II (b) nanowires used for the simulations. In the region of the junction, the diameter changes linearly with z. (c) Hysteresis loops for type-I and type-II nanowires for an applied field along the nanowire axis.
